# The c-Abl inhibitor, radotinib induces apoptosis in multiple myeloma cells via mitochondrial-dependent pathway

**DOI:** 10.1038/s41598-021-92651-9

**Published:** 2021-06-24

**Authors:** Sook-Kyoung Heo, Eui-Kyu Noh, Jeong Yi Kim, Ho-Min Yu, Jun Young Sung, Lan Jeong Ju, Do Kyoung Kim, Hye Jin Seo, Yoo Jin Lee, Jaekyung Cheon, SuJin Koh, Young Joo Min, Yunsuk Choi, Jae-Cheol Jo

**Affiliations:** 1 Biomedical Research Center, Ulsan University Hospital, University of Ulsan College of Medicine, Ulsan, 44033 Republic of Korea; 2 Department of Hematology and Oncology, Ulsan University Hospital, University of Ulsan College of Medicine, 877 Bangeojinsunhwan-doro, Dong-gu, Ulsan, 44033 Republic of Korea

**Keywords:** Biochemistry, Cancer, Cell biology, Chemical biology, Drug discovery

## Abstract

Multiple myeloma (MM) is a hematological cancer resulting from accumulated abnormal plasma cells. Unfortunately, MM remains an incurable disease, as relapse is very common. Therefore, there is urgent need to develop new treatment options for MM. Radotinib is a novel anti-cancer drug, currently approved in South Korea for the treatment of chronic myeloid leukemia patients. Its mechanism of action involves inhibition of the tyrosine kinase Bcr-Abl and the platelet-derived growth factor receptor. Generally, the mechanism of inhibition of non-receptor tyrosine kinase c-Abl has played an essential role in the inhibition of cancer progression. However, little is known regarding the effects of the c-Abl inhibitor, radotinib on MM cells. In this study, we analyzed the effect of radotinib on multiple myeloma cells. Interestingly, radotinib caused apoptosis in MM cells including RPMI-8226, MM.1S, and IM-9 cells, even in the absence of c-kit expression in 2 of these lines. Radotinib treatment significantly increased the number Annexin V-positive cells and decreased the mitochondrial membrane potential in MM cells. Additionally, we observed that cytochrome *C* was localized in the cytosol of radotinib-treated MM cells. Moreover, radotinib decreased the expression of Bcl-2 and Bcl-xL, and increased the expression of Bax and Bak in MM cells. Furthermore, radotinib promoted caspase pathway activation by inducing the expression and activity of caspase-3, -7, and -9. Expression of cleaved PARP-1 was also increased by radotinib treatment in various MM cells. In addition, radotinib significantly suppressed MM cell growth in a xenograft animal model using RPMI-8226 cells, and killed ex vivo myeloma cells from patients. In conclusion, radotinib may play an important role as a candidate agent or chemosensitizer for the treatment of MM.

## Introduction

Multiple myeloma (MM) is the second most common hematological malignancy in worldwide^1^. It is characterized by the proliferation and accumulation of malignant plasma cells in the bone marrow, and is usually associated with a monoclonal protein^2^. Anemia, infections, renal impairment or bone destruction are common clinical findings in MM patients^3,4^. Recently, there has been a dramatic increase in the number of treatment options available for the treatment of MM. In particular, the advents of immune-modulators, proteasome inhibitors, and monoclonal antibodies have led to significant improvements in the survival of patients with MM^3,5^. Unfortunately, MM remains an incurable disease, as relapse is very common. Therefore, there is a crucial need to develop novel treatment options to cure MM.

Radotinib (Supect; IY5511HCL) is an oral, multitargeted inhibitor of receptor tyrosine kinases (RTKs), including BCR-ABL, c-KIT, PDGFR, and Src family kinases^6^. A phase III trial demonstrated radotinib’s superiority over imatinib in generating a complete cytogenetic response and a major molecular response in patients with chronic phase of chronic myeloid leukemia (CML)^7^. Recently, radotinib was shown to have neuroprotective effects in a preclinical Parkinson's disease mouse model^8^. Furthermore, radotinib activated natural killer cell cytotoxicity in the Fas-expressing solid cancer cells^9^.

In our previous study, radotinib augmented cytotoxicity of the diverse AML cells^10^. Especially, it induced cell death in CD11b^+^ cells differentiated from AML blast cells^11^. Furthermore, targeting of c-KIT by radotinib promoted AML cell death in c-KIT positive AML cells^12,13^. Additionally, radotinib inhibited mitosis entry of the AML cells, and suppressed the AURKA expression in AML cells were demonstrated^14^. Generally, the mechanism of inhibition of non-receptor tyrosine kinase c-Abl has played an important role in the inhibition of cancer progression including CML^15,16^. However, little is known regarding the effects of the c-Abl inhibitor, radotinib on multiple myeloma cells. Here, we investigated the effects of radotinib in MM cells including several MM cell lines, bone marrow cells from MM patients and mouse models. Strikingly, radotinib displayed apparent antineoplastic activity in the MM cell lines and in in vivo MM xenograft models. The molecular mechanism for these results was also studied.

## Materials and methods

### Reagents

Radotinib was a generous gift from Ilyang Pharmaceutical Co., Ltd., (Seoul, South Korea) and its purity was 99.9% based on HPLC analysis^11^. All reagents were obtained from Sigma-Aldrich (St. Louis, MO, USA), unless otherwise indicated. The CellTiter 96 AQueous One Solution Cell Proliferation Assay was purchased from Promega (Madison, WI, USA). Apoptosis Detection Kit I was obtained from BD Bioscience (San Diego, CA, USA). NE-PER Nuclear and Cytoplasmic Extraction Reagents was obtained from Thermo Fisher Scientific (Waltham, MA, USA). The antibodies used in western blot analysis, anti-cytochrome *C*, Bcl-2, and β-actin were purchased from Santa Cruz Biotechnology (Santa Cruz, CA, USA), while the rest were purchased from Cell Signaling Technology (Beverly, MA, USA). TUNEL Assay Kit was obtained from Abcam (San Diego, CA, USA). CasGLOW Fluorescein Active Caspase-3 and Caspase-9 Staining Kit were purchased from Thermo Fisher Scientific (Waltham, MA, USA).

### Ethics approval

All human-related methods were carried out in accordance with relevant guidelines and regulations. All patients were given written informed consent prior to study commencement, and written informed consent was obtained from all patients. The study protocol and patient consent form and information were approved by the Ulsan University Hospital Ethics Committee and Institutional Review Board (UUH-IRB-2016-07-026). All procedures involving animals were in accordance with the Laboratory Animals Welfare Act, the Guide for the Care and Use of Laboratory Animals, and the Guidelines and Policies for Rodent Experimentation provided by the Institutional Animal Care and Use Committee (IACUC) of the Ulsan University of Korea. This study protocol was approved by the institutional review board of the Ulsan University of Korea (Approval No. 0118-02).

### Patient samples

All patients were newly diagnosed with MM (NDMM, *n* = 14), at Ulsan University Hospital, Ulsan, South Korea, as described in Supplementary Table 1. Bone marrow samples were collected before administering the first round of chemotherapy. In addition, the bone marrow samples of patients with bortezomib (velcade) and dexamethasone refractory multiple myeloma were also collected (VD refractory MM, *n* = 9).

### Cell culture

The RPMI-8226, MM.1S, and IM-9 cells were obtained from the American Type Culture Collection (ATCC, Manassas, VA, USA). These cells were grown as suspension in RPMI 1640 medium supplemented with 10% heat-inactivated FBS and 1% penicillin–streptomycin in a 5% CO_2_ humidified atmosphere at 37 °C.

### Isolation of patient cells and culture

The patient cells were isolated by the density gradient method, as previously described^17^. In brief, bone marrow cells (BMCs) were isolated via density gradient centrifugation at 400×*g* using Lymphoprep (Axis-Shield, Oslo, Norway). They were washed with phosphate-buffered saline (PBS) and cultured with RPMI1640 with 10% FBS and 1% penicillin–streptomycin in a 5% CO_2_ humidified atmosphere at 37 °C.

### Cell viability assay

Cell viability was measured by the CellTiter 96 AQueous One Solution Cell Proliferation Assay (Promega, Madison, WI, USA), according to the manufacturer's protocol. Briefly, cells were seeded in a 96-well plate with 200 μl of medium per well at a density of 3 × 10^4^ cells/ml, and then incubated with 0, 10, 50 and 100 μM of radotinib for 72 h at 37 °C. Analysis was performed by adding CellTiter 96 solution to each well, and the plate was incubated for an additional 4 h in a humidified 5% CO_2_ atmosphere at 37 °C. Finally, absorbance was measured at 490 nm using a SpectraMax iD3 microplate reader (Molecular Devices, San Jose, CA, USA).

### Cell surface staining of CD38

Cell surface staining was performed with anti-human CD38-PE-CY5 (Clone HIT2, BD bioscience, San Diego, CA, USA) and isotype control mAb (mouse IgG-PE-CY5), as previously described^11^. The cells were analyzed using a FACSCalibur flow cytometer and CellQuest Pro software (BD Bioscience).

### Detection of Annexin V positive cells

RPMI-8226, MM.1S, and IM-9 cells were seeded in 24-well plates at a density of 3 × 10^4^ cells/well, and then incubated with 0, 10, 50, and 100 µM of radotinib for 72 h at 37 °C, harvested and washed twice with FACS buffer (PBS containing 0.2% FBS and 0.1% NaN3). Then, the cells were stained with Annexin V-FITC from the Apoptosis Detection Kit I, according to the manufacturer's instructions. Cells were analyzed using the FACSCalibur flow cytometer and CellQuest Pro software (BD Biosciences, CA, USA).

### Analysis of mitochondrial membrane potential

Multiple myeloma cells were incubated with 0, 10, 50, and 100 µM of radotinib for 72 h at 37 °C, harvested and washed twice with PBS buffer. Measurement of the mitochondrial membrane potential (MMP, *ΔΨ*_*m*_) was performed as previously described^10^.

### Measurement of caspase-3 and -9 activities

Cells were incubated with various concentrations of radotinib (0, 10, 50, and 100 µM) for 72 h at 37 °C. Then, cells were evaluated using the CasGLOW Fluorescein Active Caspase-3 and Caspase-9 Staining Kit according to the manufacturer's instructions (Thermo Fisher Scientific, MA, USA).

### Western blotting analysis and immunoprecipitation

Western blotting analysis and immunoprecipitation were conducted under the same conditions as above, and were performed as previously described^10,13^. In some experiments, cytosolic fraction of cells was extracted using the NE-PER Nuclear and Cytoplasmic Extraction reagents according to the manufacturer's instructions (Thermo Fisher Scientific, MA, USA). The blots were developed using the ChemiDoc Touch Imaging System, and analyzed with the Image Lab Software, Version 6.0.1, https://www.bio-rad.com/en-kr/SearchResults?MaxResults=10&Text=image%20lab%20software&Page=1&TabName=SOFTWARETYPE&FieldName=SOFTWARETYPE,CATEGORYLEVEL0,PRODUCT_TYPE,LITERATURETYPE,DIVISIONNAME,SKULEVEL0,WEBTYPE, Bio‑Rad Laboratories, CA, USA).

### Mice

Specific-pathogen-free 5-week-old athymic nude male mice were purchased from OrientBio (Seongnam, Korea), and kept in a clean environment of the Ulsan University of Korea (Korea, Ulsan). All mice (2–4 mice/cage) were housed under standard conditions at constant temperature and humidity, food and water were freely available, and were treated according to the IACUC of the University of Ulsan (Ulsan, Korea, Approval No. 0118-02).

### Xenograft animal model using RPMI-8226 cells

The study was carried out in compliance with the ARRIVE guidelines. We performed the experiment in the same way as the xenograft animal model using acute myeloid leukemia cells, as previously described^13,18^. If detailed protocol is required, please refer to the above references. In briefly, the athymic nude male mice were implanted with RPMI-8226 cells (3 × 10^7^ cells/mouse) in the right flank region (*n* = 5 for each group). Once tumors were established, mice were treated with vehicle or 100 mg/kg ip radotinib daily every 5/7 day for up to 24 day. Radotinib treatment was performed intraperitoneally for 5 days except weekends. The number of mice used in each experiment is described in Supplementary Table 2. The tumors were excised, and each tumor tissue homogenized for the preparation of cell samples for several analyses.

### TUNEL assay

Tumor tissue samples were evaluated for apoptosis to determine DNA double strand breaks using the TUNEL Assay Kit according to the manufacturer's instructions, as previously described^18^.

### Statistics

Experimental results were analyzed including calculation of mean and standard error (mean ± SEM) using ImageJ (version 1.5, LOCI, Madison, WI, USA) and GraphPad Prism 7.0 (GraphPad Software, La Jolla, CA, USA). Differences among multiple means were assessed by one-way ANOVA or two-way ANOVA followed by Tukey’s test or by Bonferroni’s multiple comparisons test as appropriate. Probabilities of *P* < 0.05 were considered statistically significant.

## Results

### The c-Abl inhibitor, radotinib induces apoptosis in multiple myeloma cells

**Figure 1:**
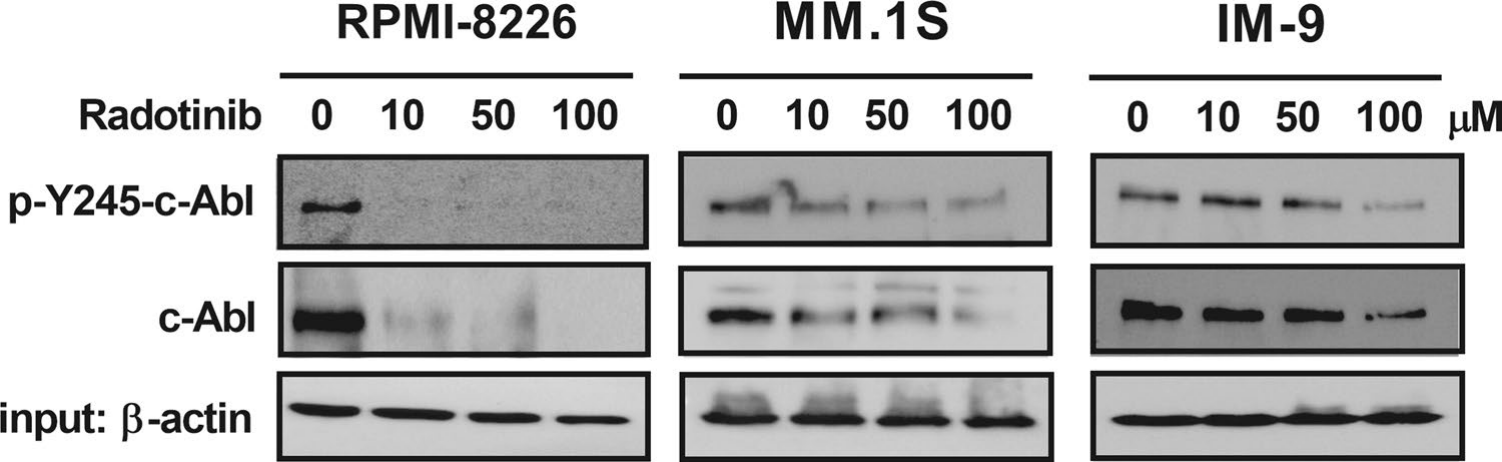
Radotinib reduces activation and expression of the c-Abl in MM cells, including RPMI-8226, MM.1S, IM-9. Cells were incubated with 0, 10, 50, and 100 μM radotinib for 72 h. The c-Abl activation and expression by radotinib were confirmed by immunoprecipitation analysis.

The mechanism of inhibition of non-receptor tyrosine kinase c-Abl has played an essential role in the inhibition of cancer progression^15,16^. Radotinib reduced activation and expression of the c-Abl in MM cells, including RPMI-8226, MM.1S, IM-9. Cells were incubated with 0, 10, 50, and 100 μM radotinib for 72 h. And then the c-Abl activation and expression by radotinib were confirmed by immunoprecipitation analysis. According to Fig. 1, treatment with radotinib reduced activation and expression of the c-Abl in MM cells. These results showed that radotinib could act as the c-Abl inhibitor in multiple myeloma cells. At the same time, those results were shown that the effect of radotinib on MM cells targets c-Abl, not an off-target effect.

**Figure 2:**
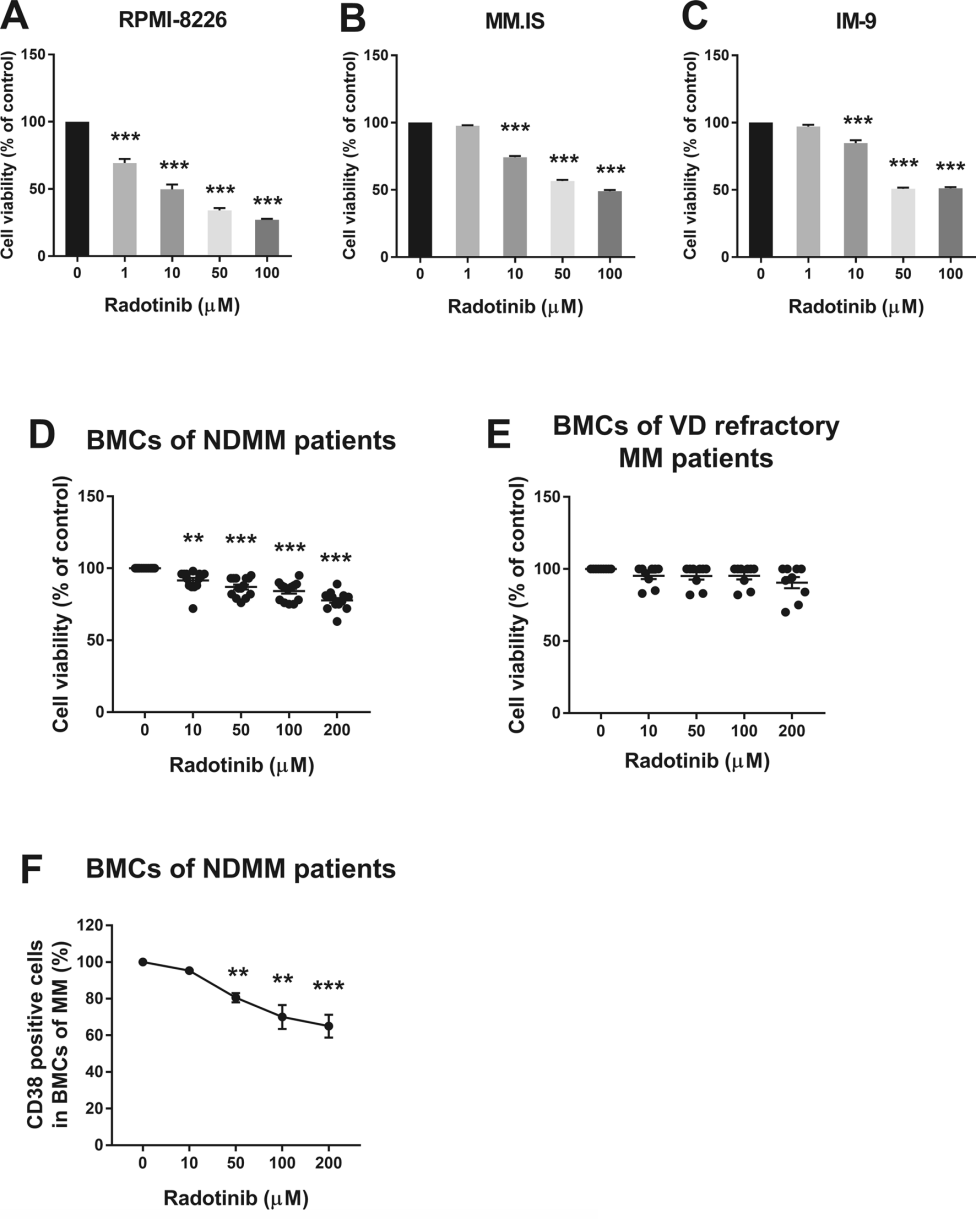
Effects of radotinib on the viability of multiple myeloma cells. Cells were incubated with 0, 1, 10, 50, and 100 μM radotinib for 72 h in RPMI-8226 (**A**), MM.1S (**B**), and IM-9 cells (**C**). BMCs of MM patients (*n* = 14) and BMCs of VD refractory MM patients (*n* = 9) were incubated with 0, 10, 50, 100 and 200 μM radotinib for 72 h (**D**,**E**). BMCs were collected and processed under the same conditions as described above (**F**). Then CD38 positive cells were monitored in BMCs of MM patients (*n* = 5). Data are presented as mean ± SEM.; **P < 0.01; ***P < 0.001. *Significantly different from control cells. BMCs, Bone marrow cells; NDMM, Newly diagnosed multiple myeloma; VD refractory MM, Bortezomib (velcade) and dexamethasone refractory multiple myeloma.

The structure of radotinib is shown in Supplementary Fig. 1. To investigate the effect of radotinib on the growth of MM cells, RPMI-8226, MM.1S, and IM-9 cells were treated with 0, 1, 10, 50, and 100 μM radotinib for 72 h and cell viability was measured by the CellTiter 96 solution. As shown in Fig. 2A–C, radotinib exerted a significant cytotoxic effect on the three types of MM cell lines in a dose-dependent manner (IC_50_ in 72 h, RPMI-8226: 10 µM, MM.1S: 100 µM, and IM-9: 100 µM). These results indicate that radotinib had cytotoxic effects on multiple myeloma cells.

MM cells depend on bone marrow stroll or stromal cells to survive and proliferate^19^. Most of the currently available drugs should be evaluated for their activity against MM cells from the patients with multiple myeloma. In addition, it is very important that the activity of radotinib in MM cells and also in bortezomib/dexamethasone refractory MM cells. In this regard, we have tested radotinib cytoxicity in bone marrow cells (BMCs) of patients with multiple myeloma. BMCs of newly diagnosed multiple myeloma (NDMM) patients and BMCs of bortezomib (velcade) and dexamethasone refractory multiple myeloma (VD refractory MM) patients were incubated with 0, 10, 50, 100 and 200 μM radotinib for 72 h. As expected, cytotoxicity of radotinib was observed in BMCs of NDMM patients, as shown in Fig. 2D. Radotinib cytotoxicity on cell viability measurements was observed in all 14 of the 14 BMCs samples from NDMM patients (BMCs death in NDMM patients at 72 h, 100 µM: 15%; 200 µM: 23%; with the presence of BMSC). And radotinib cytoxicity was observed in 3 of the 9 BMC samples of VD refractory MM patients in the cell viability measurement, and 6 samples were unresponsive (Fig. 2E).

In addition, BMCs were collected under the same conditions as described above. Then the expression of CD38 positive cells, well known as the cell membrane antigen of MM, monitored in both BMCs of MM patients and VD refractory MM patients. The reason for monitoring CD38 in particular is that it is important to observe the trend of multiple myeloma cells in the bone marrow cells. The expression of CD38 positive cells was significantly decreased in BMCs of NDMM patients (Fig. 2F).

**Figure 3:**
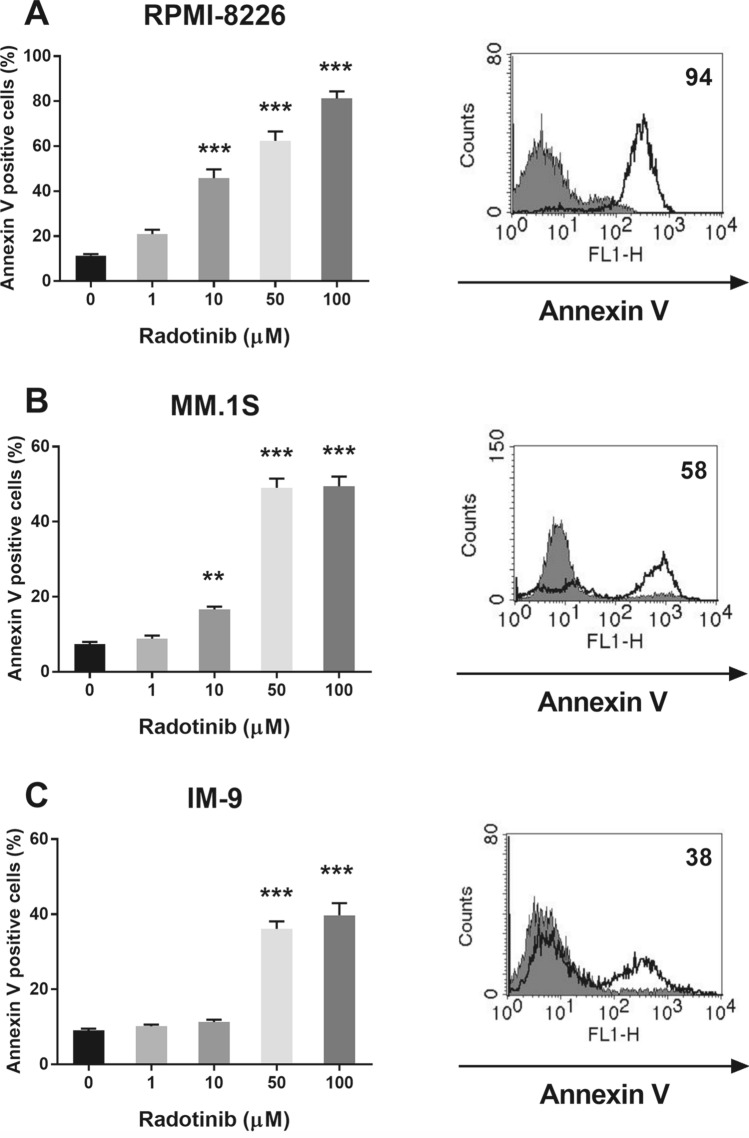
Radotinib induced apoptosis in MM cells. The cells were also collected and treated under the same conditions described above. Cells were stained with Annexin V, followed by flow cytometry analysis. Radotinib induced dose-dependent apoptosis in RPMI-8226 (**A**), MM.1S (**B**) and IM-9 cells (**C**). The filled histogram represents Annexin V positive cells treated with 0 µM radotinib (control group), and the open histogram represents Annexin V positive cells treated with 100 µM radotinib, respectively. Data are presented mean ± SEM.; **P < 0.01.; ***P < 0.001. *Significantly different from control cells.

Next, we examined the ability of radotinib to induce apoptosis in MM cell lines. The cells were also cultured and treated under the same conditions as described above. Cells were stained with Annexin V-FITC, followed by flow cytometric analysis. As shown in Fig. 3A–C, radotinib increased the number of apoptotic cells in a dose-dependent manner in RPMI-8226, MM.1S, and IM-9 cell cultures. Therefore, these results suggest that radotinib induces apoptosis in MM cells (Fig. 3).

### Radotinib induces mitochondrial- and caspase-dependent apoptosis in RPMI-8226, MM.1S and IM-9 cells

**Figure 4:**
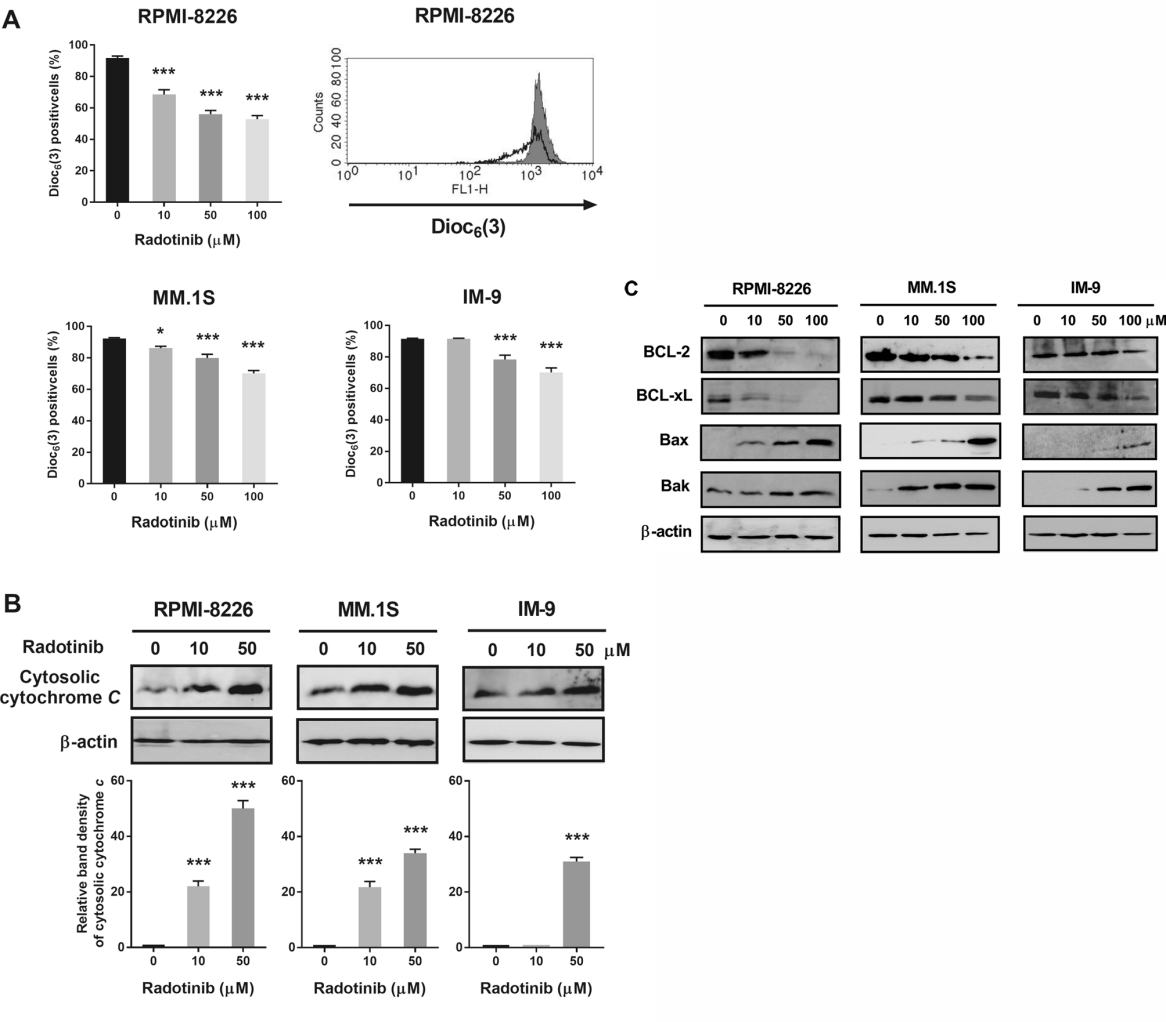
Radotinib-induced apoptosis by activation of the mitochondrial pathway. RPMI-8226, MM.1S, and IM-9 cells were incubated with 0, 10, 50, and 100 μM radotinib for 72 h. (**A**) Effect of radotinib on mitochondrial membrane potential (MMP, *ΔΨm*). Filled histogram indicates MMP in cells treated with 0 μM radotinib, and open histogram indicates MMP in RPMI-8226 cells treated with 100 μM radotinib. (**B**) Effect of radotinib on cytosolic cytochrome *C* levels. (**C**) Effects of radotinib on the expression of BCL-2 family proteins (including BCL-2, BCL-xL, Bax, and Bak). The membrane was stripped and reprobed with anti-β-actin mAb to confirm equal loading. Data present mean ± SEM.; *P < 0.05.; ***P < 0.001. *Significantly different from control cells.

**Figure 5:**
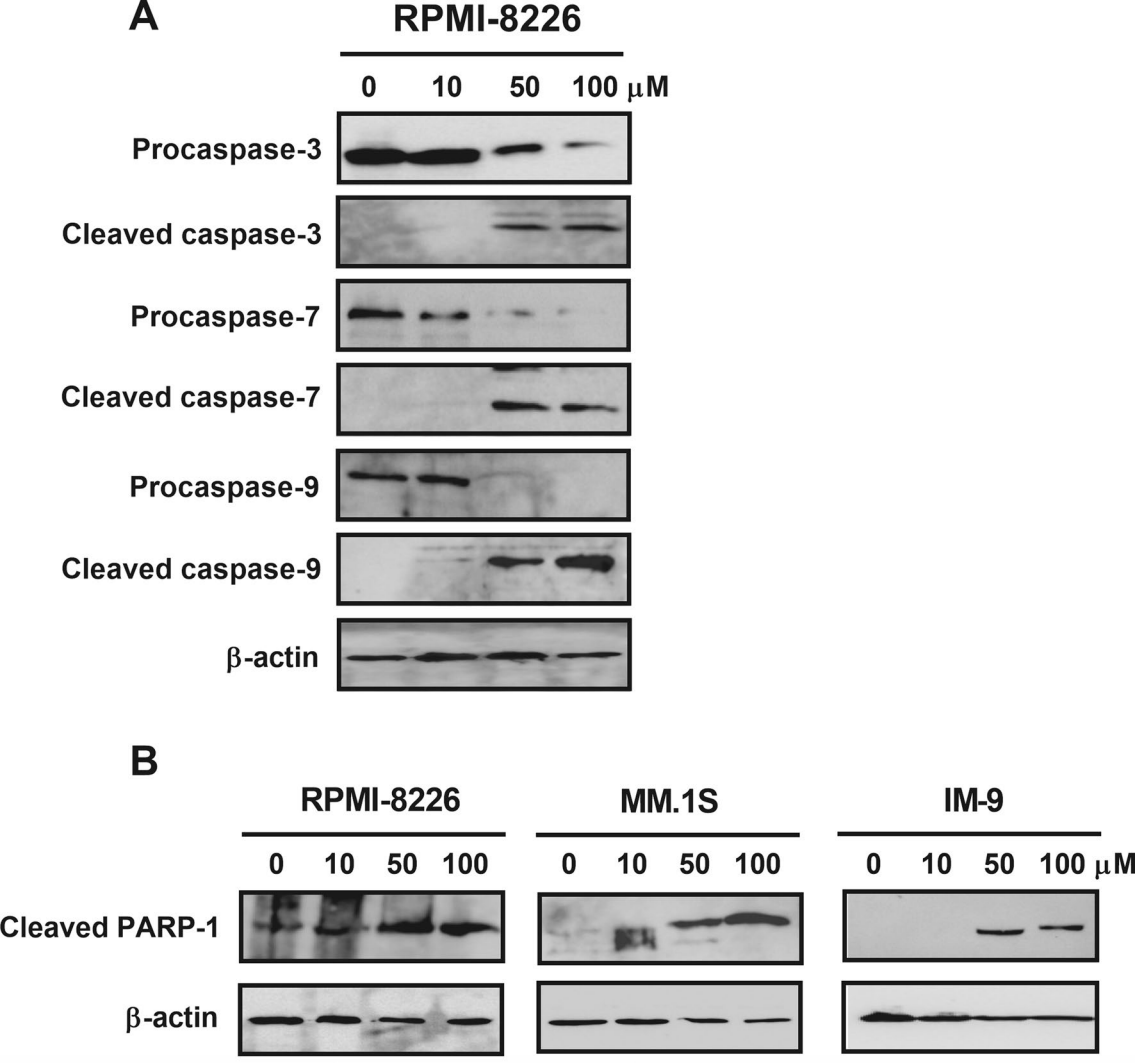
Radotinib activates the caspase pathway in MM cells. The expression of caspase-3, -7, -9 (**A**), and cleaved PARP-1 (**B**) was measured by western blot analysis. Data are representative of three independent experiments.

**Figure 6:**
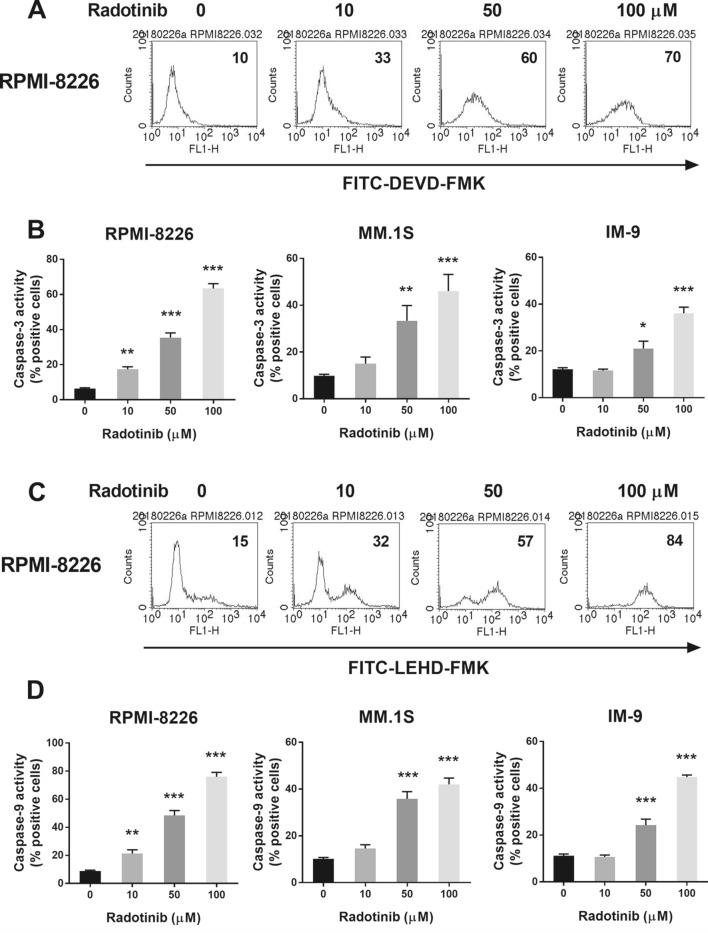
Radotinib activates caspase-3 and caspase-9 activities in MM cells. RPMI-8226, MM.1S, and IM-9 cells were incubated with 0, 10, 50, and 100 μM radotinib for 72 h. Caspase-3 and caspase-9 activities were measured as described in “Materials and methods”. (**A**,**B**) Caspase-3 activity in MM cells. (**C**,**D**) Caspase-9 activity in MM cells.

Stimulation of the intrinsic apoptotic pathway is a crucial mechanism through which most chemotherapeutic agents induce cancer cell death. This pathway involves dysfunction of mitochondria and activation of caspases^20^. Mitochondria play a very significant role in apoptosis^21^. Herein, we examined the effect of radotinib on mitochondrial membrane potential (MMP, *ΔΨ*_*m*_) in MM cells by using the DiOC_6_(3) dye. Radotinib treatment for 72 h remarkably decreased MMP in RPMI-8226, MM.1S, and IM-9 cells (Fig. 4A). Translocation of cytochrome *C* from the mitochondria to the cytosol is important for the activation of caspases and eventually apoptosis^22,23^. In the present study, we observed that cytochrome *C* accumulated in a dose-dependent manner in the cytosol following treatment of the three cell lines with radotinib (Fig. 4B). Moreover, radotinib decreased the expression of BCL-2 and BCL-xL, while it increased the expression of Bax and Bak in RPMI-8226, MM.1S, and IM-9 cells (Fig. 4C). These data indicate that radotinib induces apoptosis through the mitochondrial pathway (Fig. 4). Mostly, caspase-3 plays a vital role in the terminal phase of apoptosis^22^. We observed that expression of procaspase-3, procaspase-7, and procaspase-9 levels was decreased by radotinib in a dose-dependent manner. In addition, radotinib treatment augmented the levels of cleaved caspase-3, caspase-7, and caspase-9 levels in RPMI-8226 cells (Fig. 5A). Finally, the expression of cleaved PARP-1 was significantly enlarged in RPMI-8226, MM.1S and IM-9 cells following treatment with radotinib (Fig. 5B). Furthermore, caspase-3 and caspase-9 activities increased more than 7- and 5.5-fold, respectively, with increasing radotinib concentrations in RPMI-8226 cells (Fig. 6A–D). In addition, caspase-3 and caspase-9 activities were similarly increased following radotinib treatment in MM.1S and IM-9 cells. These results show that radotinib induces mitochondria- and caspase-dependent apoptosis in MM cells (Figs. 3, 4, 5, 6).

### Radotinib significantly suppressed MM cell growth in a xenograft animal model using RPMI-8226 cells

**Figure 7:**
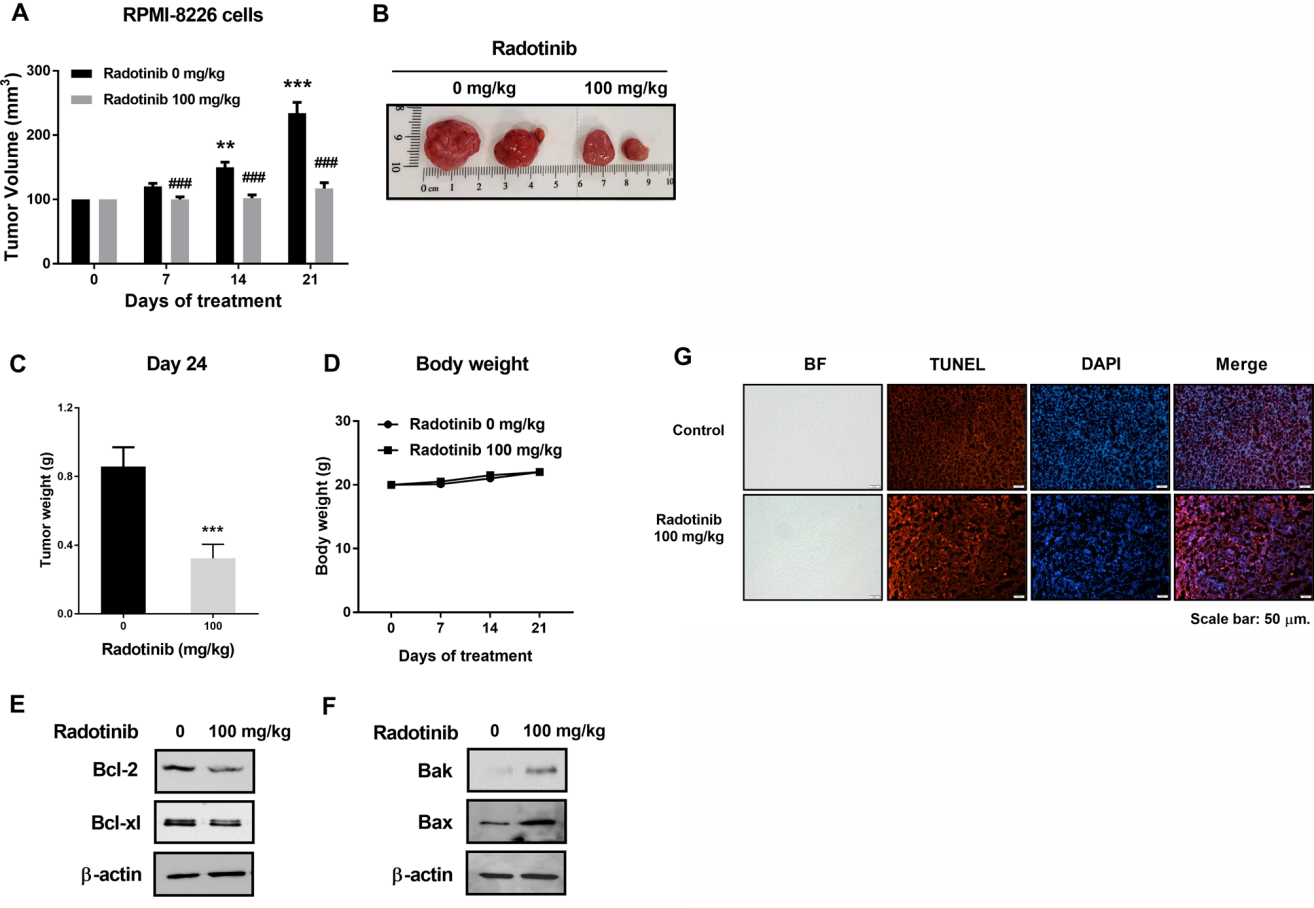
Radotinib significantly suppressed MM cell growth in a xenograft animal model (*n* = 5 for each group). (**A**) Tumor volume (mm^3^). (**B**) Representative photographs of tumors 24 days after radotinib treatment. (**C**) Tumor weight. (**D**) Body weight of mouse. (**E**,**F**) The expression of BCL-2, BCL-xL, Bak, and Bax in RPMI-8226 cells isolated from the tumor tissue. The membrane was also incubated with anti-β-actin mAb to confirm equal loading. (**G**) Measurement of DNA double-strand breaks in tumor tissue. Radotinib significantly increased the DNA double-strand breaks in tumor tissue. TUNEL assay was used for measurement of DNA double-strand breaks in tumor tissue as described in “Materials and methods”. Upper panel, radotinib 0 mg/kg; lower panel, radotinib 100 mg/kg. Results are representative of three independent experiments. Data are presented as mean ± SEM. Significantly different from the day 0 control (*) or each day control (#); (*); **P < 0.01; ***, ^###^*P* < 0.001.

To further validate the molecular mechanism of radotinib in vivo, we established murine models of multiple myeloma. Radotinib restrained the growth of xenografted RPMI-8226 cells in nude mice (Fig. 7A–C). After confirming the anti-myeloma effect of radotinib, all mice were euthanized, and survival curves were drawn and analyzed, as shown in the Supplementary Fig. 2. The body weight of the tumor-bearing mice did not change significantly during the duration of this study, as shown in Fig. 7D. In addition, radotinib inhibited expression of Bcl-2, Bcl-xl, Bak, and Bax in RPMI-8226 cells isolated from the tumor tissue (Fig. 7E,F). Furthermore, DNA double-strand breaks in RPMI-8226 cells isolated from tumor tissue were measured by a TUNEL assay using the fragmentation marker dUTP, following treatment with 0 mg/kg and 100 mg/kg radotinib. Radotinib significantly increased the number of DNA double-strand breaks in the tumor tissue (Fig. 7G). These results are similar to the results obtained using the Annexin V staining of RPMI-8226 cells. Once again, these results suggest that radotinib significantly inhibits MM cell growth in vivo and may potentially be used for the treatment of MM in the future. Collectively, the data highlight the therapeutic possibility of radotinib in MM cells (Fig. 7).

### Radotinib enhances boterzomib/dexamethasone-induced MM cell death

**Figure 8:**
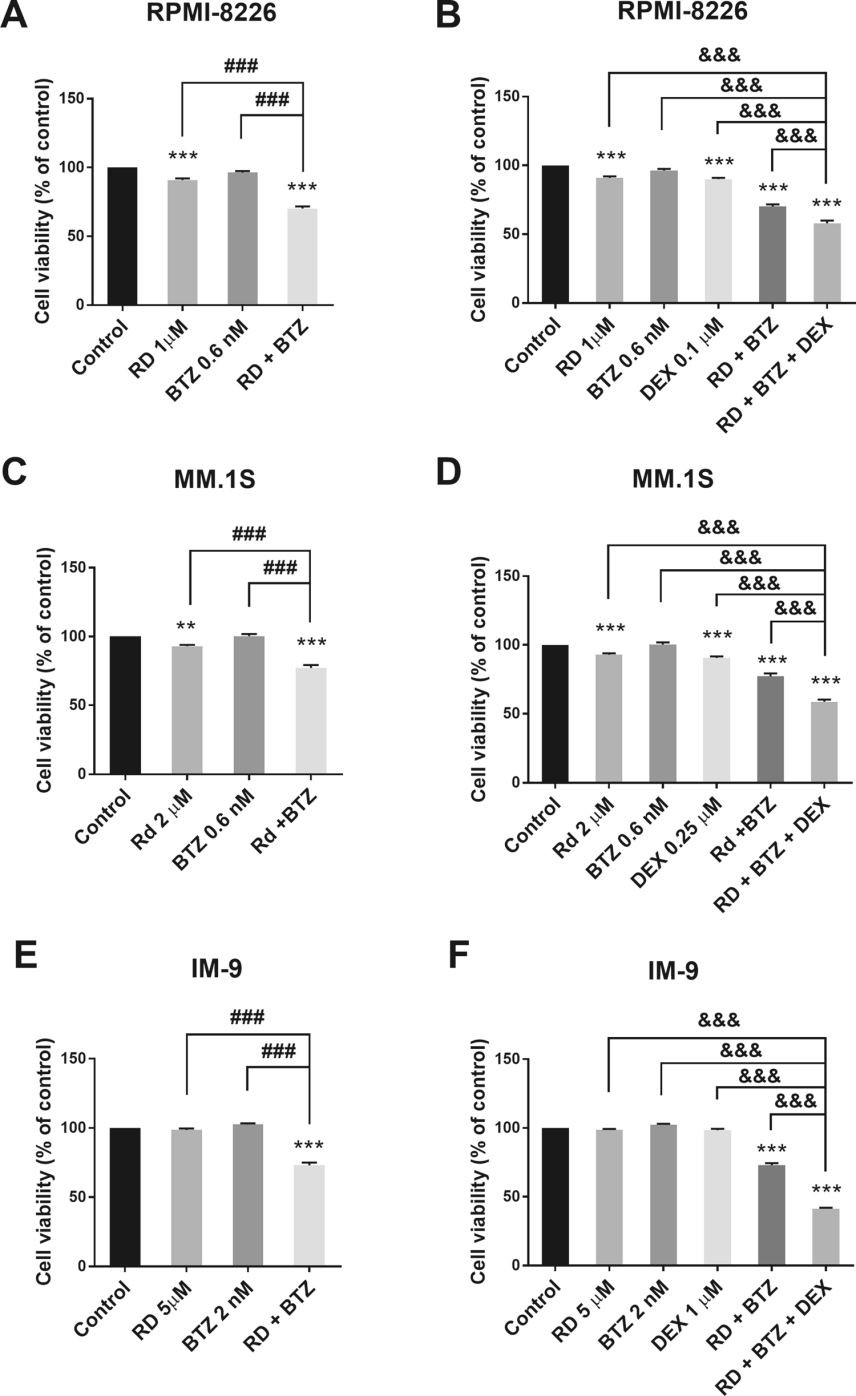
Triple combination effects of radotinib, bortezomib and dexamethasone on cell viability assay in multiple myeloma cell lines, RPMI-8226, MM.1S and IM-9 cells. Cell viability assay was measured by CellTiter 96 solution assay kit (Promega, WI, USA). Combination treatment results for 48 h with radotinib and bortezomib in RPMI-8226 (**A**), MM.1S (**C**) and IM-9 cells (**E**). Triple combination treatment results for 48 h with radotinib, bortezomib and dexamethasone in RPMI-8226 (**B**), MM.1S (**D**) and IM-9 cells (**F**). These data represent the means ± SEM. Significantly different from the control (*), combination (#) and triple combination (&); ***, ^###^, ^&&&^*P* < 0.001. RD, radotinib; BTZ, bortezomib, DEX, dexamethasone; RD + BTZ, Combination of radotinib and bortezomib; RD + BTZ + DEX, Triple combination of radotinib, bortezomib and dexamethasone.

First of all, we researched the combination effects of radotinib and conventional chemotherapeutic agent for multiple myeloma, and bortezomib. We already confirmed the cytotoxicity on combination of radotinib and chemotherapeutic agent, bortezomib in diverse MM cells including RPMI-8226, MM.1S, and IM-9 cells (Fig. 8A,C,E). And synergistic effects of two on the apoptotic proteins were observed in our results (Supplementary Fig. 3).

In addition, we examined the combination effects of radotinib and conventional chemotherapeutic agents for multiple myeloma, bortezomib and dexamethasone. We confirmed the cytotoxicity on triple combination of radotinib, bortezomib and dexamethasone in diverse MM cells including RPMI-8226, MM.1S, and IM-9 cells (Fig. 8B,D,F). Therefore, radotinib could be helpful to treat the MM disease. Thus, we expect that radotinib might be a potential drug for Anti-MM therapy in MM. In particular, even low concentrations of radotinib may enhance susceptibility of MM cells to death by boterzomib and dexamethasone, as shown in Fig. 8.

## Discussion

Apoptosis has been a well-known as the target of cancer therapeutic strategies^24^. In addition, apoptosis is a type of programmed cell death that is fundamental for cancer treatment^25,26^. Activation of the intrinsic apoptotic pathway is an important mechanism through which most chemotherapeutic agents induce cancer cell death. Generally, the pathway involves mitochondria, caspase-dependent activation, and so on^20,27,28^.

MM is a malignant disease characterized by accumulation of terminally differentiated plasma cells in the bone marrow^4,29^. According to classical protocols, treatment of MM is dependent on few drugs, such as lenalidomide, bortezomib, and dexamethasone. Recently, there are the number of available options for the treatment of MM such as immunomodulating agents, proteasome inhibitors, monoclonal antibodies and inhibitor of nuclear export^5^. However, MM still recurs easily and is difficult to be cured. Therefore, there is a crucial need for identifying novel therapeutic options for MM.

Radotinib is being used for the treatment of different types of cancer. It is approved in South Korea for its use as a second-line treatment of CML. Its mechanism of action involves inhibition of the tyrosine kinase Bcr-Abl and of platelet-derived growth factor receptor. Little is known regarding the effects of radotinib on MM cells. Interestingly, radotinib caused death of MM cells (Fig. 1). Radotinib increased the number of Annexin V positive cells, indicating that cells undergo death via apoptosis (Fig. 2). Furthermore, radotinib treatment remarkably decreased MMP levels and resulted in a dose-depended accumulation of cytochrome *C* in the cytosol of RPMI-8226, MM.1S, and IM-9 cells. Moreover, radotinib decreased the expression of Bcl-xL and Bcl-2, and increased the expression of Bax and Bak in MM cells (Figs. 3, 4). In addition, caspases-3, -7, and -9 were activated (Figs. 5, 6). These results indicate that radotinib induces death of MM cells by induction of mitochondrial- and caspase-dependent apoptosis. Generally, the mitochondrial apoptotic pathway is known as the upstream stage of the caspase apoptotic pathway. To summarize our results in one sentence, radotinib induces apoptosis in multiple myeloma cells via mitochondrial-dependent pathway.

To further validate the anti-myeloma effects of radotinib in vivo, we established xenograft murine models using the human multiple myeloma cell line RPMI-8226. We confirmed that radotinib had anti-tumor activities in mice bearing RPMI-8226 cells (Fig. 7). In addition, radotinib significantly increased DNA double-strand breaks in the tumor tissue (Fig. 7G). Additionally, radotinib enhanced boterzomib/dexamethasone-induced MM cell death (Fig. 8). Collectively, the data obtained in this study highlight the therapeutic potential of radotinib in MM cells. Therefore, radotinib may play an important role as a candidate drug or chemosensitizer for the treatment of MM.

Radotinib, a c-Abl inhibitor, induces apoptosis in multiple myeloma cells through a mitochondrial-dependent pathway. According to our results, the degree of apoptosis response by radotinib differed among multiple myeloma cell lines. For example, RPMI-8226 cells were very sensitive to radotinib and less sensitive in to IM-9 cells. And MM.1S cells were in the middle. Above all, it is very essential to study which molecules were targeted by radotinib. As shown in Fig. 1, radotinib decreased activation and expression of c-Abl in MM cells including RPMI-8226, MM.1S, and IM-9. Therefore, we basically thought it was important to check the expression level of the target molecules against radotinib in the multiple myeloma cell lines, RPMI-8226, MM.1S, and IM-9 cells. We measured the relative expression levels of c-Abl and c-Kit in RPMI-8226, MM.1S, and IM-9 cells by Western blotting. As shown in Supplementary Figure 4A, c-Abl, which is considered to be a target molecule of radotinib, was expressed in all RPMI-8226, MM.1S and IM-9 cells. In particular, relative expression of c-Abl was high in the RPMI-8226 cells. Interestingly, however, the expression of non-receptor tyrosine kinase, c-Abl was very high in RPMI-8226 cells, which has a high level of apoptosis. And the cell death was the lowest in IM-9 cells with the lowest c-Abl expression. Therefore, these results indicate that the higher the c-Abl expression, the higher the cell death by radotinib. On the other hand, c-Kit, which is considered to be another target molecule of radotinib, was expressed at high levels in only RPMI-8226 cells (Supplementary Figure 4A,B). Remarkably, the expression of c-Kit and was very high in RPMI-8226, which has a high level of apoptosis. Therefore, more research is needed on these results, but the present results suggest that the expression levels of c-Abl and c-Kit, and MM cell death might be correlated. In addition, the expression of RTK, PDGFRβ was not expressed in all the cells including RPMI-8226, MM.1S, IM-9 (Supplementary Figure 4C).

We have shown through previous studies that dasatinib and radotinib induced high cytotoxicity in c-Kit positive AML cells. At the same time, dasatinib and radotinib induce reduction of c-Kit expression in KAUMI-1 and HEL92.1.7 cells. Therefore, c-Kit suppression by dasatinib and radotinib was essential for AML cell death via apoptotic pathway activation. Moreover, cells with high c-Kit expression induced higher cell death by dasatinib and radotinib. Additionally, c-Kit internalization by dasatinib and radotinib caused c-Kit positive AML cell death^13^. Considering these points, results for a high level of cell death of c-Kit positive MM cells, RPMI-8226, was expected. These results showed that the cytotoxicity of radotinib in c-Kit positive cells could be maximized but also demonstrate that c-Kit expression was not required for activity in 2 of the 3 c-abl^+^ cell lines.

Even though no kinase inhibitors have received FDA approval for MM therapy, preclinical exploration for possible therapeutic targets in MM has yielded the potential results, and small molecule protein kinase inhibitors have been under investigation in its application to MM^30^. We confirmed that the antitumor activity of radotinib that significantly inhibited and delayed MM tumor growth in vitro and vivo (Figs. 1, 2, 3, 4, 5, 6, 7). According to our results, radotinib enhanced BTZ-induced MM cell death in diverse cell lines (Fig. 8). Moreover, combined treatment with radotinib and BTZ had a synergistic effect on RPMI-8226 cell viability (combination index: 0.24, Supplementary Fig. 5). In addition, radotinib enhanced boterzomib/dexamethasone-induced MM cell death (Fig. 8). Taken together, the data obtained in this study highlight the therapeutic potential of radotinib in MM cells. Thus, radotinib may play an important role as a candidate drug or chemical sensitizer for the treatment of MM. We summarize the results of this study in Supplementary Fig. 6. In this regard, research into the mechanism of action of radotinib is valuable as this compound may be used for the treatment of MM. We plan to further investigate whether this compound has anti-cancer activity against c-Abl, c-Kit or other targets in the future. In addition, combination treatment with radotinib and BTZ or DEX was only performed in vitro, to make it more clinically relevant, so further studies in the xenograft models are needed to validate these results.

Many treatment options for patients with MM are still under investigation. Most patients eventually experience serial relapse and are treated with most available agents at some point during their disease course. Moreover, a preferred order for their use has not been established. A variety of novel drugs are being studied for the treatment of MM including immune-modulating drugs, proteasome inhibitors, monoclonal antibodies, HDAC inhibitors, nuclear export inhibitor and so on^5^. The combination treatment with above drug leads to additive and/or synergistic effects. Therefore, we think that small progress through various translational and clinical researches is the basis for the development of multiple myeloma treatment. In this context, our findings with radotinib in MM cell lines could also contribute to the development of MM treatment research. In this respect, we think that it is a very valuable study.

In conclusion, radotinib induces apoptosis in MM cells by inducing the mitochondria- and caspase-dependent pathway. Therefore, our results indicate that radotinib can abrogate MM cell growth both in vitro and in vivo and may serve as a candidate agent or a chemosensitizer for the treatment of MM. In addition, radotinib may be used in combination with other chemotherapeutic agents such as bortezomib or dexamethasone for MM treatment. This study provides the first evidence that the c-Abl inhibitor, radotinib could be used as an effective therapeutic agent to treat MM as it induces mitochondria- and caspase-dependent apoptosis.

## Supplementary Information

Supplementary Information.

## Data Availability

The datasets used and/or analyzed during the current study are available from the corresponding author on reasonable request.
