# Supplementary material for: The c-Abl inhibitor, radotinib induces apoptosis in multiple myeloma cells via mitochondrial-dependent pathway
Source: Sci Rep. 2021 Jun 24;11:13198. doi: 10.1038/s41598-021-92651-9 (PMC8225673; doi:10.1038/s41598-021-92651-9)
Supplement: Supplementary file 1 — Supplementary Information. [file 41598_2021_92651_MOESM1_ESM.docx]

**Supplementary Materials**

**The c-Abl inhibitor, Radotinib induces apoptosis in multiple myeloma cells via mitochondrial-dependent pathway**

Sook-Kyoung Heo, Eui-Kyu Noh, Jeong Yi Kim, Ho-Min Yu, Jun Young Sung, Lan Jeong Ju, Do Kyoung Kim, Hye Jin Seo, Yoo Jin Lee, Jaekyung Cheon, SuJin Koh, Young Joo Min, Yunsuk Choi, Jae-Cheol Jo

**Supplementary Table 1. Characteristics of patient’s derived samples**

|  |  | **NDMM** | **Bortezomib refractory**  **MM** |
| --- | --- | --- | --- |
|  |  | **N = 14** | **N = 9** |
| **Median age,**  **years (range)** |  | 66  (51-80) | 75  (54-84) |
| **Gender (*n*)** | **Female** | 7 | 6 |
|  | **Male** | 7 | 3 |
| **FISH (*n*)** | **Standard** | 6 | 4 |
|  | **High** | 6 | 1 |
|  | **NA** | 2 | 4 |
| **ISS (*n*)** | **I** | 4 | 0 |
|  | **II** | 4 | 5 |
|  | **III** | 6 | 4 |
| **Median previous no. of therapies** |  | 0 | 2 (1-4) |

*NDMM* Newly diagnosed multiple myeloma, *ISS* revised international staging system, *NA* not assessed Fluorescence in situ hybridization (FISH), was categorized as standard and high risk. Standard risk included normal cytogenetics and hyperdiploidy, t(11;14), t(6;14), 13q deletion and hypodiploidy, while high risk included t(4;14), 17p deletion, and t(14;16).

**
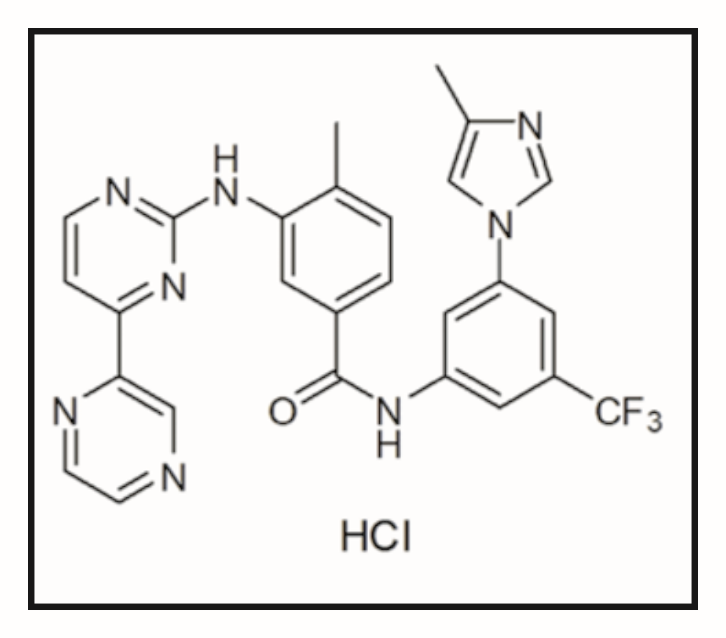
**

**Supplementary Figure 1**. The structure of radotinib.

**
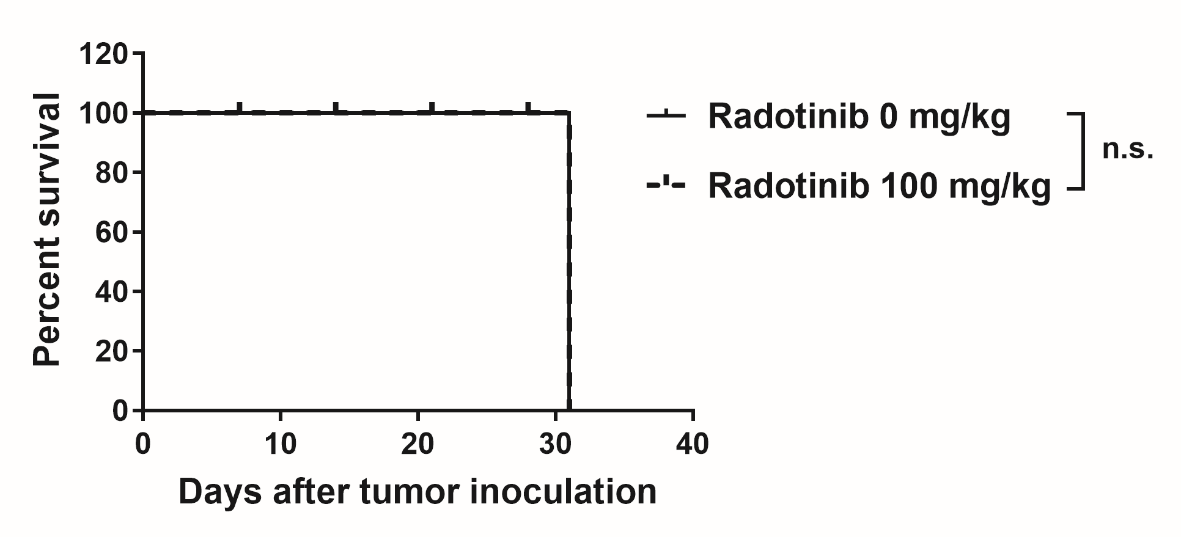
**

**Supplementary Figure 2.** Kaplan–Meier survival curve. n.s., not significant, log-rank (Mantel–Cox) test for significance.

**
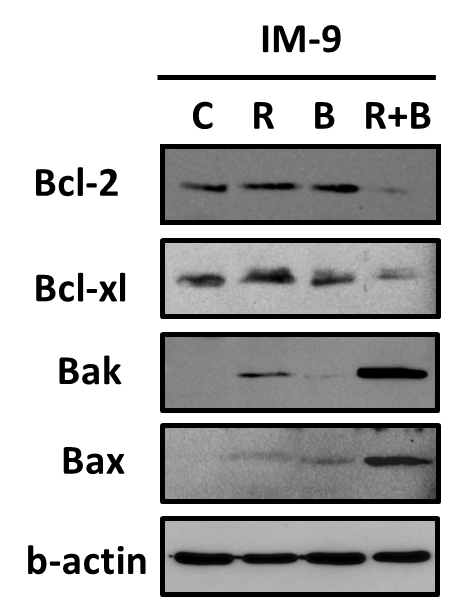
**

**Supplementary Figure 3**. Combination effects of radotinib and chemotherapeutic agent, bortezomib on the expression of BCL-2 family proteins (including BCL-2, BCL-xL, Bax, and Bak) in IM-9 cells. Cells were incubated with 5 μM radotinib and 2 nM for 48 h in IM-9 cells. Expression of Bcl-2, Bcl-xl, Mcl-1, Bak and Bax in IM-9 cells by radotinib and bortezomib was measured by Western blotting analysis. The membrane was also incubated with anti-β-actin mAb to confirm equal loading. Results are representative of three independent experiments. R, radotinib; B, bortezomib; R + B, Combination of radotinib and bortezomib.


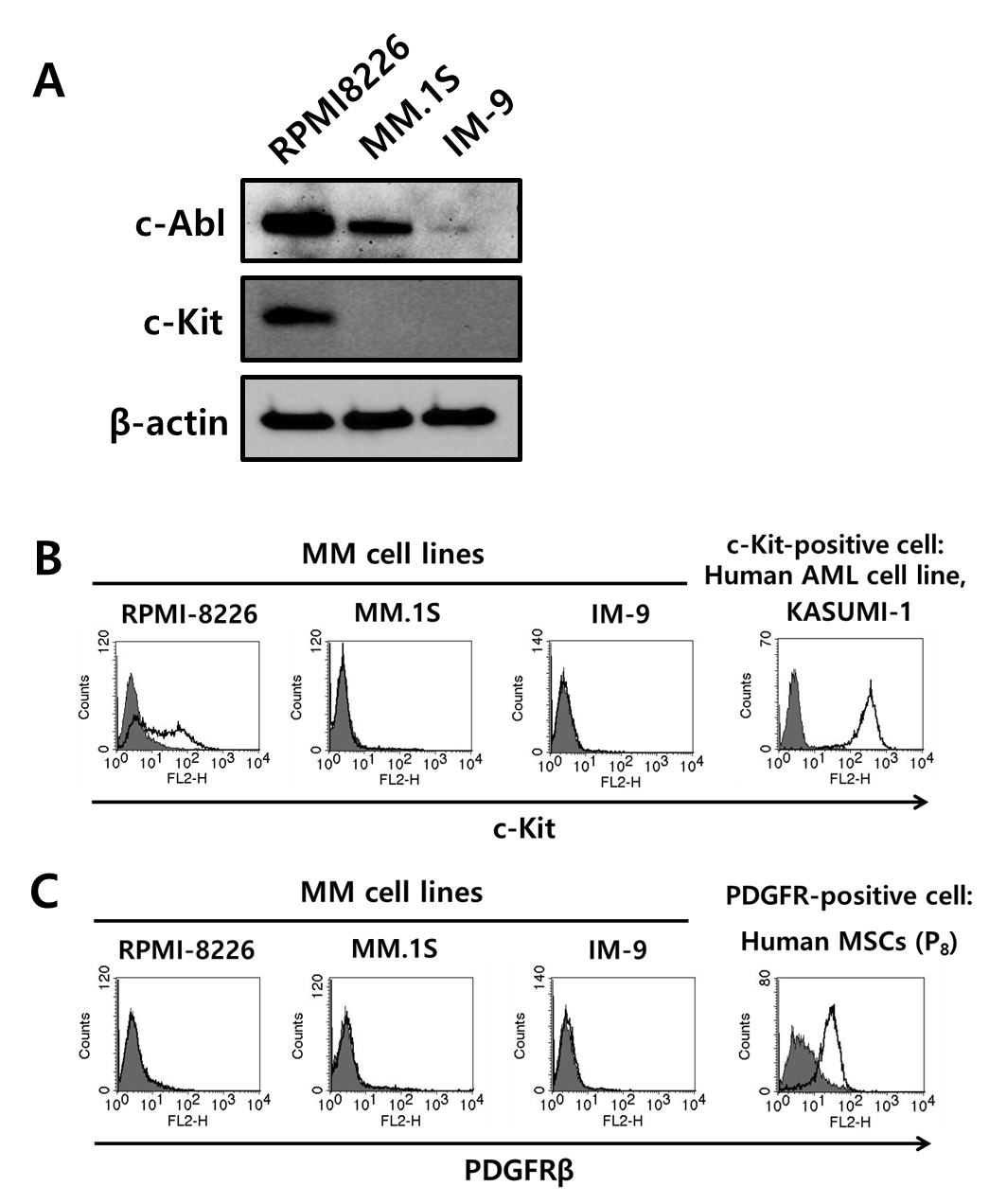


**Supplementary Figure 4.** The relative expression levels of c-Abl and c-Kit in RPMI-8226, MM.1S, and IM-9 cells were analyzed by Western blotting (A). The membrane was stripped and reprobed with anti-β-actin mAb to confirm equal loading. Expression of receptor tyrosine kinases, c-Kit and PDGFRβ in RPMI-8226, MM.1S, and IM-9 cells (B, C). Cell surface staining was performed with anti-human c-Kit-PE (BD bioscience, San Diego, CA, USA), anti-human PDGFRβ-PE (e-bioscience, Atlanta, GA, USA), and each isotype control mAbs. The filled histogram represents the isotype control, and the open histogram represents each target (c-Kit and PDGFR) positive cells. Kasumi-1 cells and human mesenchymal stem cells (passage, 8) are positive controls for each antigen.





**Supplementary Figure 5.** **Isobologram analysis of radotinib and BTZ combination on MM cell death.** Cell viability assay by radotinib and BTZ was analyzed in RPMI-8226 cells. Cells were seeded (density, 5 × 10^4^ cells/well) in 96-well plates containing 200 µl medium per well and were incubated with diverse concentration of radotinib and/or BTZ for 48 h at 37°C. CellTiter 96 solution (20 µl; Promega, Madison, WI, USA) was added directly to each well, and the plates were incubated for 4 h in a humidified atmosphere of 5% CO_2_ at 37°C. Absorbance was measured at 490 nm by using SpectraMax iD3 Microplate Reader (Molecular Devices, San Jose, CA, USA). We found the strong synergism on radotinib and BTZ combination on MM cell death. Fifty % of inhibition concentration (IC_50_) on AML cell death in HL60 cells: Radotinib only, 25 μM; BTZ only, 2 nM; combination of radotinib and BTZ = 1 μM + 0.4 nM. Combination Index: 0.24).


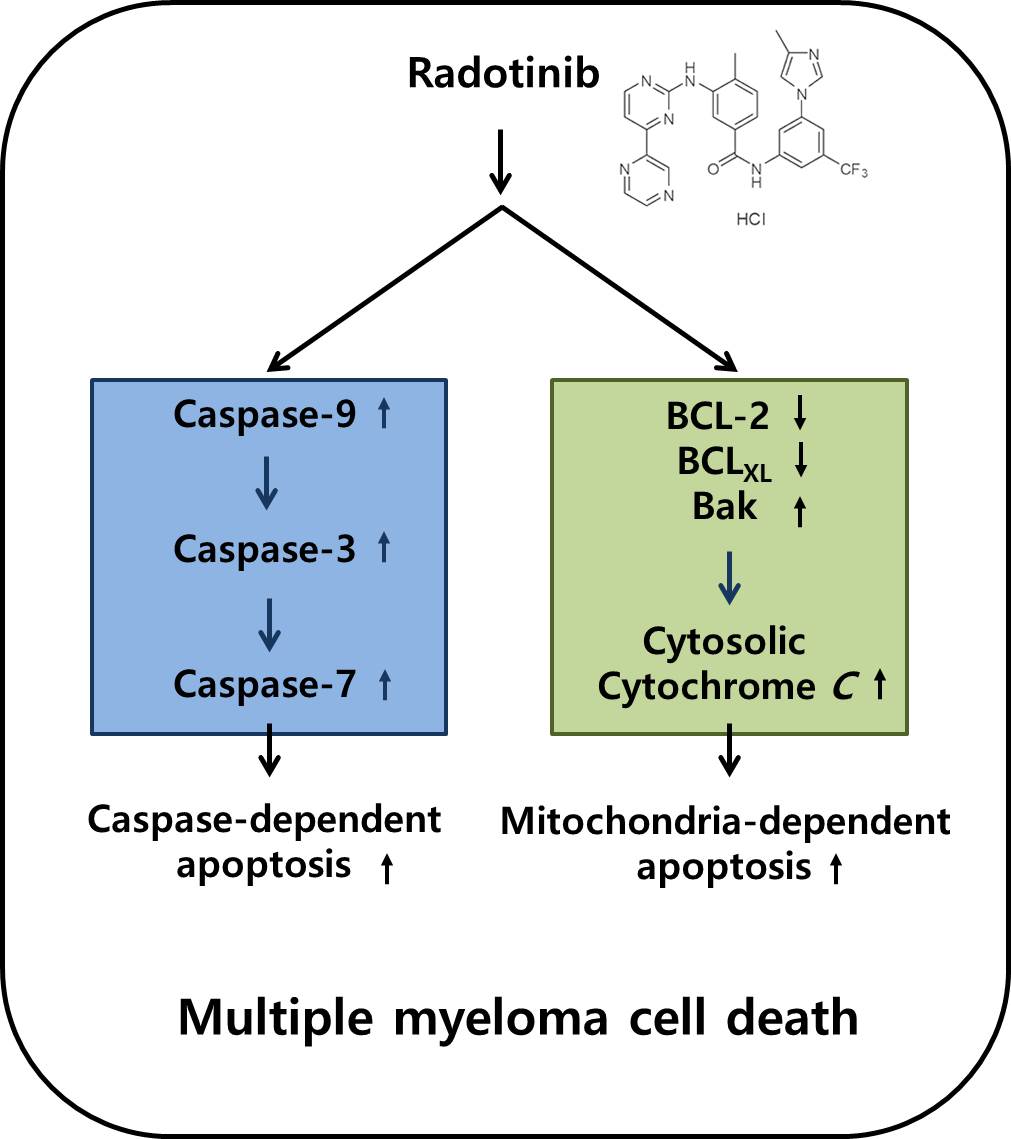


**Supplementary Figure 6**. Proposed pathway of Radotinib in human MM cells. Radotinib caused cell death of MM cells. Radotinib induced Annexin V positive cells. Treatment with radotinib remarkably decreased MMP in MM cells. Cytochrome *C* accumulated dose-dependently in the cytosol of radotinib-treated RPMI-8226, MM.1S, and IM-9 cells. Moreover, radotinib decreased the expression of Bcl-xL and Bcl-2, and increased the expression of Bax and Bak in MM cells. Additionally, the caspase pathway was activated including caspase-3, -7, and -9. Moreover, radotinib significantly suppressed MM cell growth in a xenograft animal model. Radotinib may play an important role as a candidate agent or chemosensitizer for the treatment of MM.

**
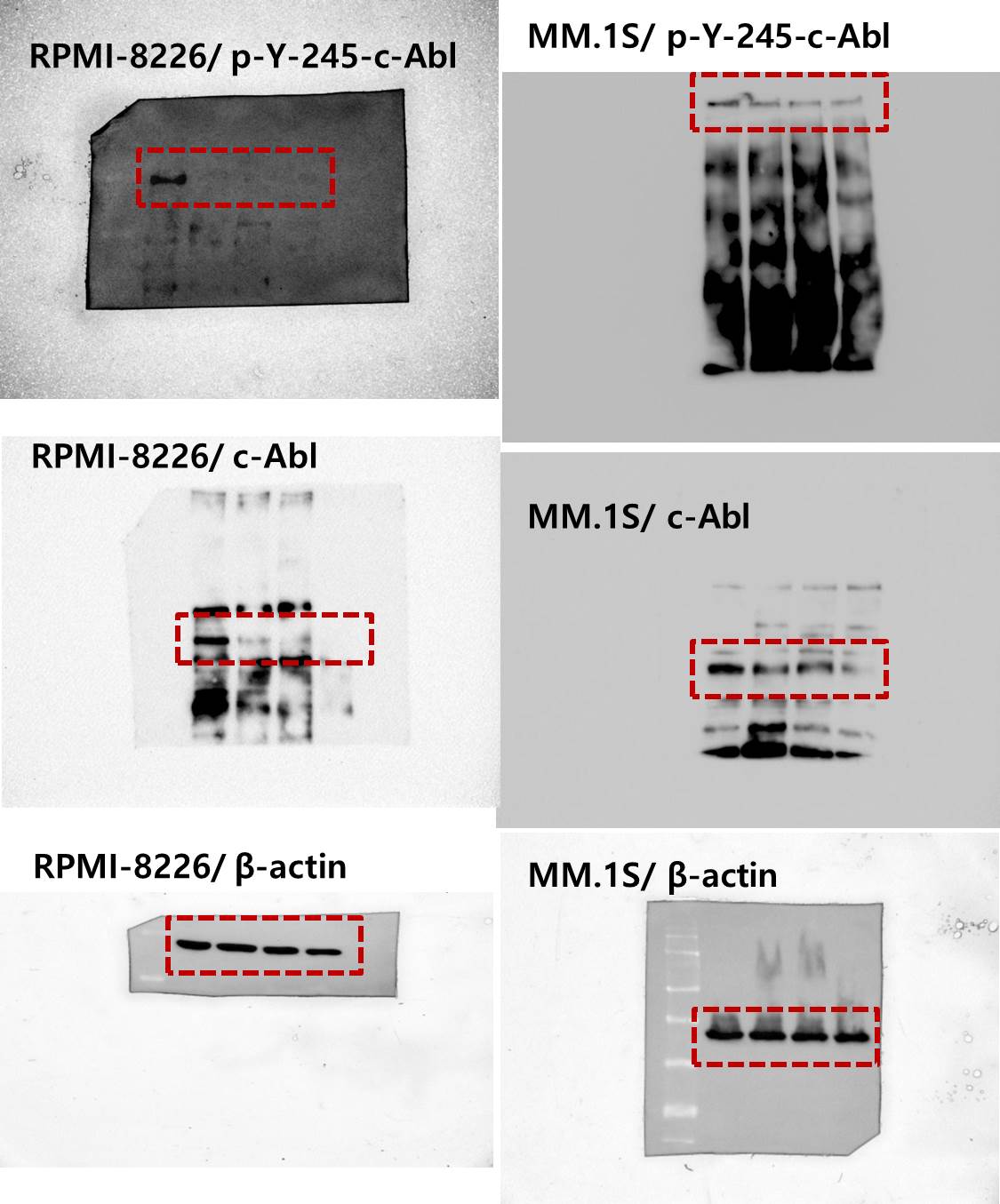
**

**Supplementary Figure 7.** Original western blots used for **Fig. 1**. The blots were developed using the ChemiDoc Touch Imaging System, and analyzed with the Image Lab Software. The red boxes indicate the cropped regions used in the representative figures.

**
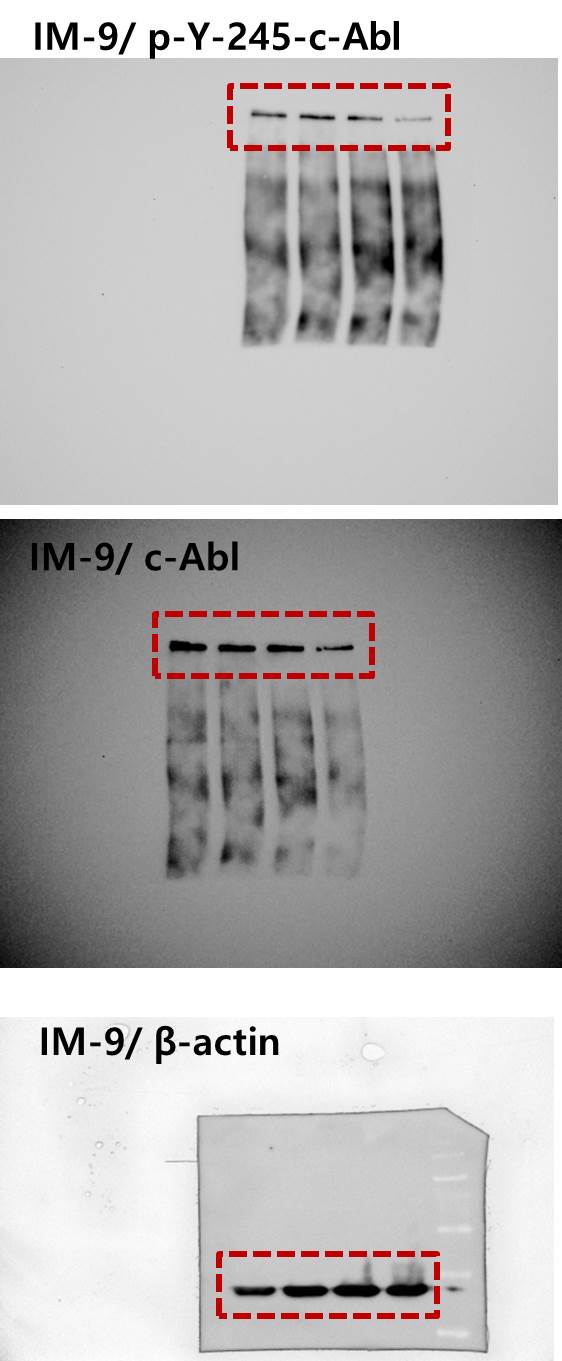
**

**Supplementary Figure 7. continued**

**
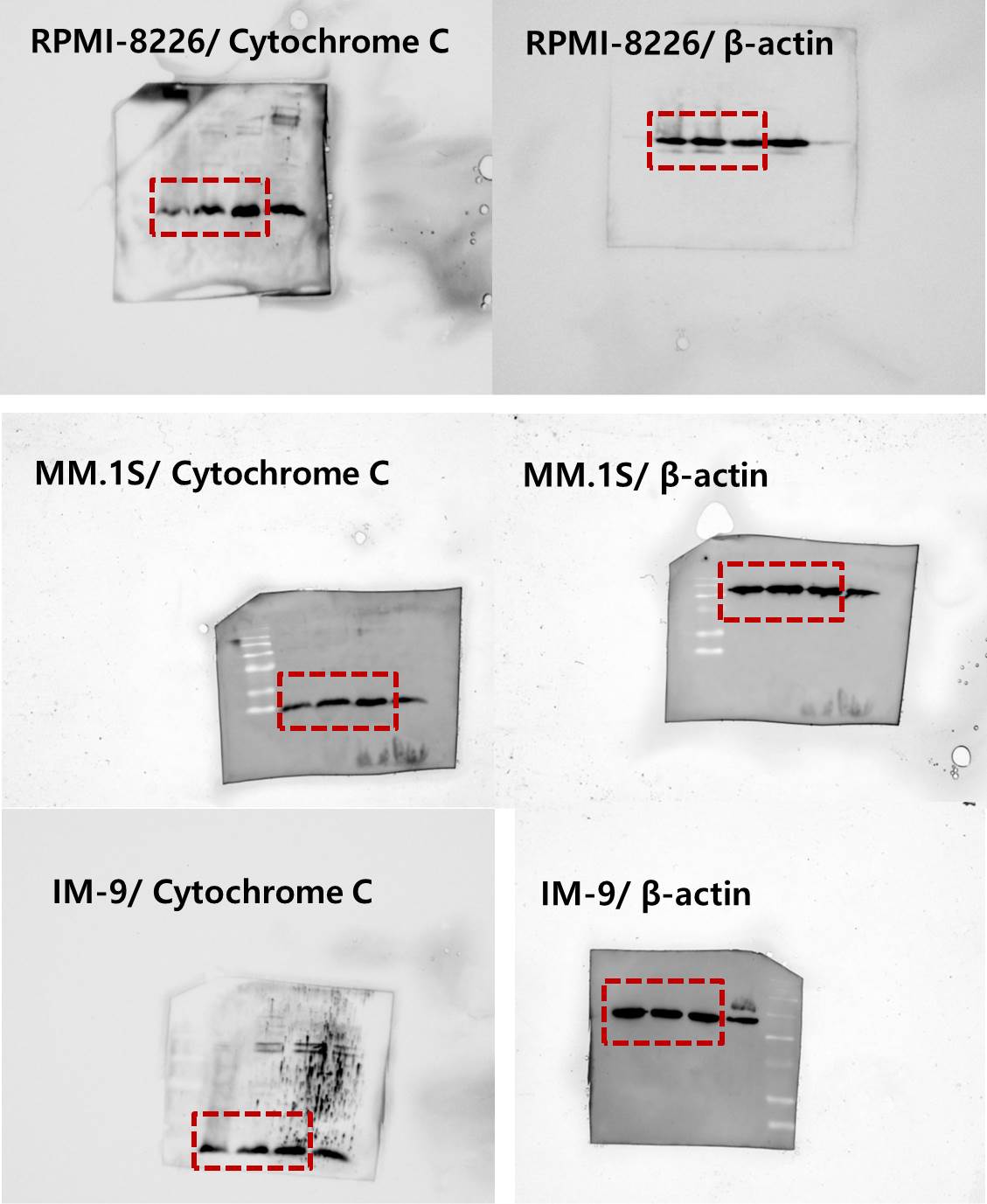
**

**Supplementary Figure 8.** Original western blots used for **Fig. 4B and 4C**. The blots were developed using the ChemiDoc Touch Imaging System, and analyzed with the Image Lab Software. The red boxes indicate the cropped regions used in the representative figures.


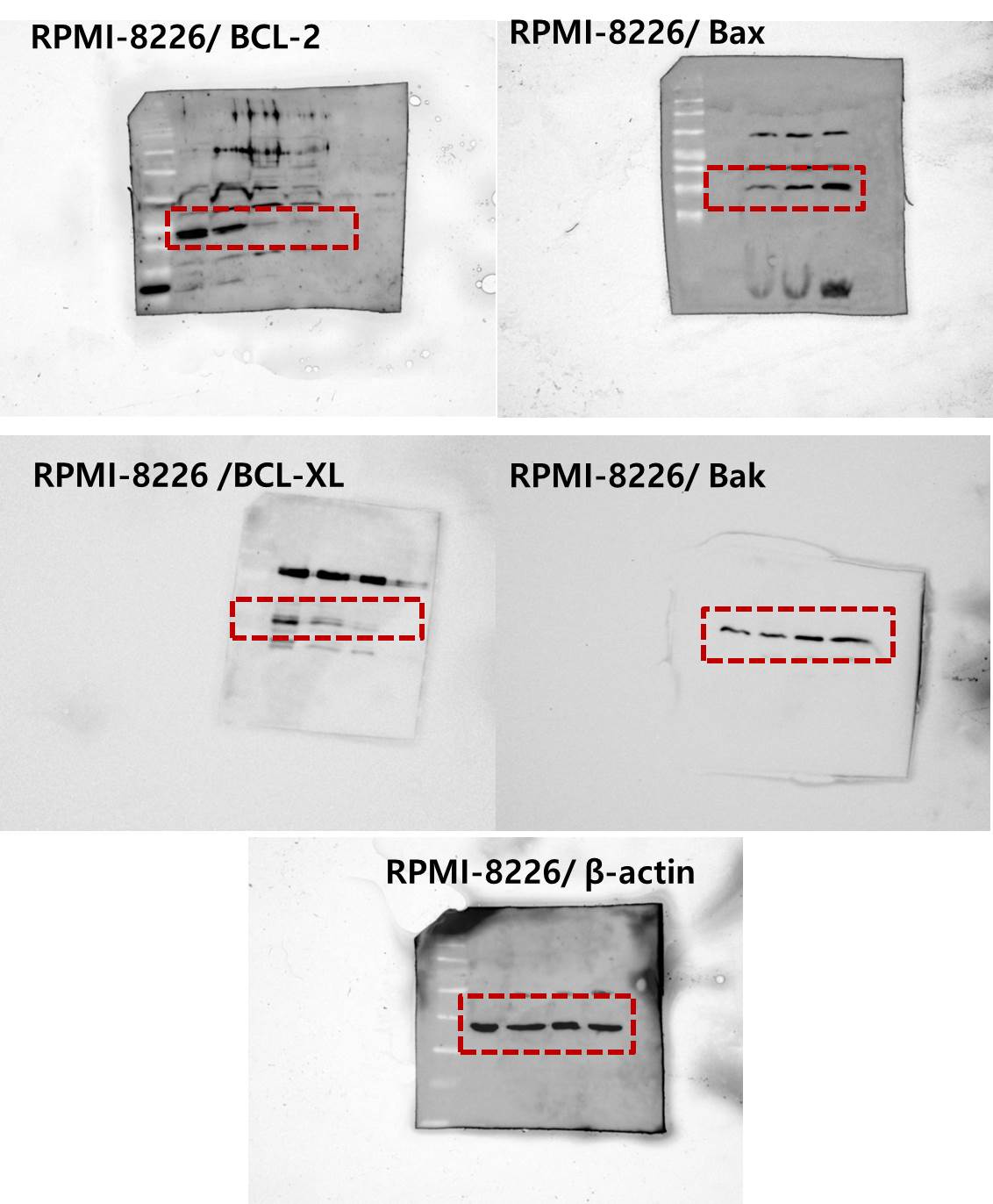


**Supplementary Figure 8. continued**

**
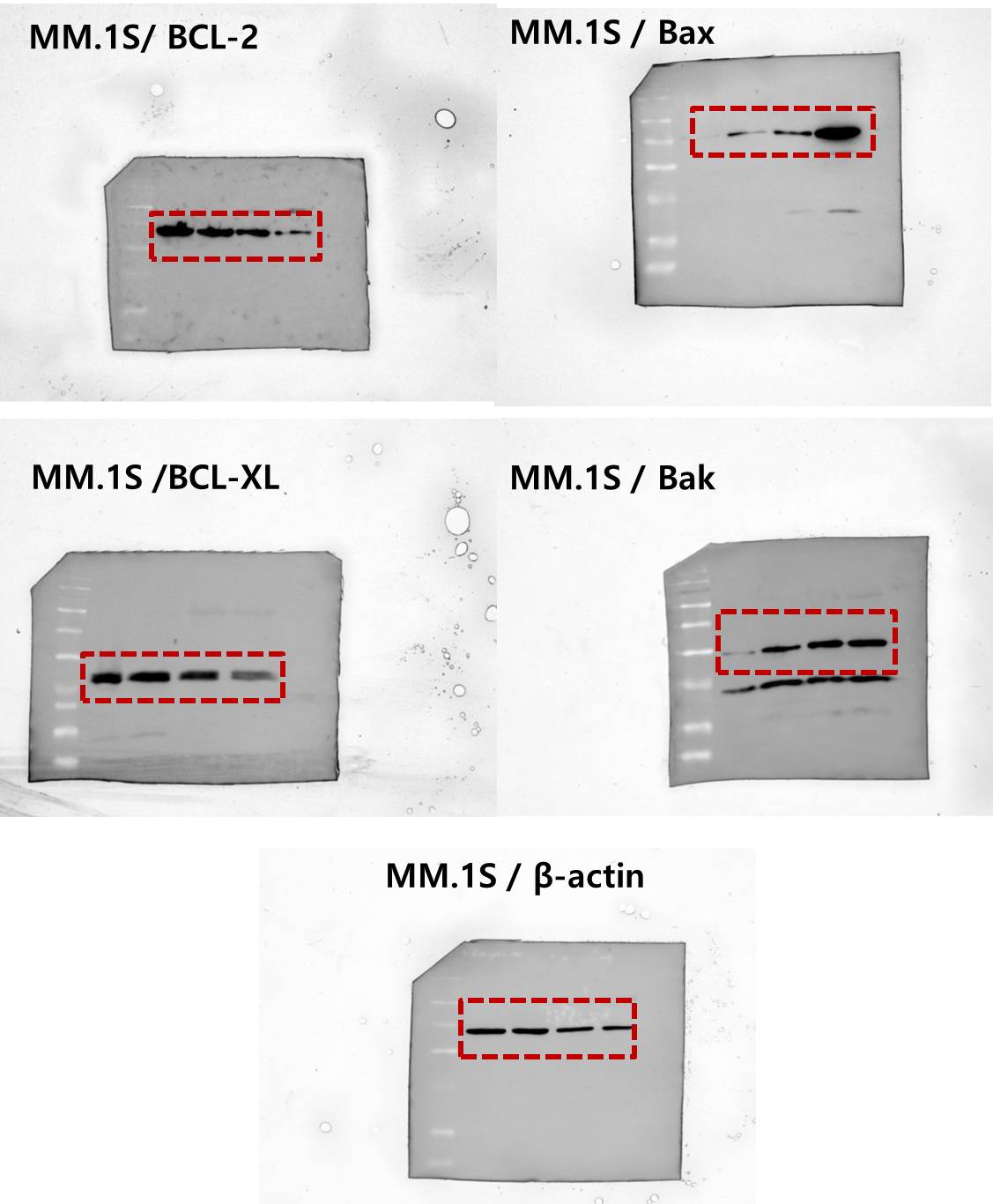
**

**Supplementary Figure 8. continued**

**
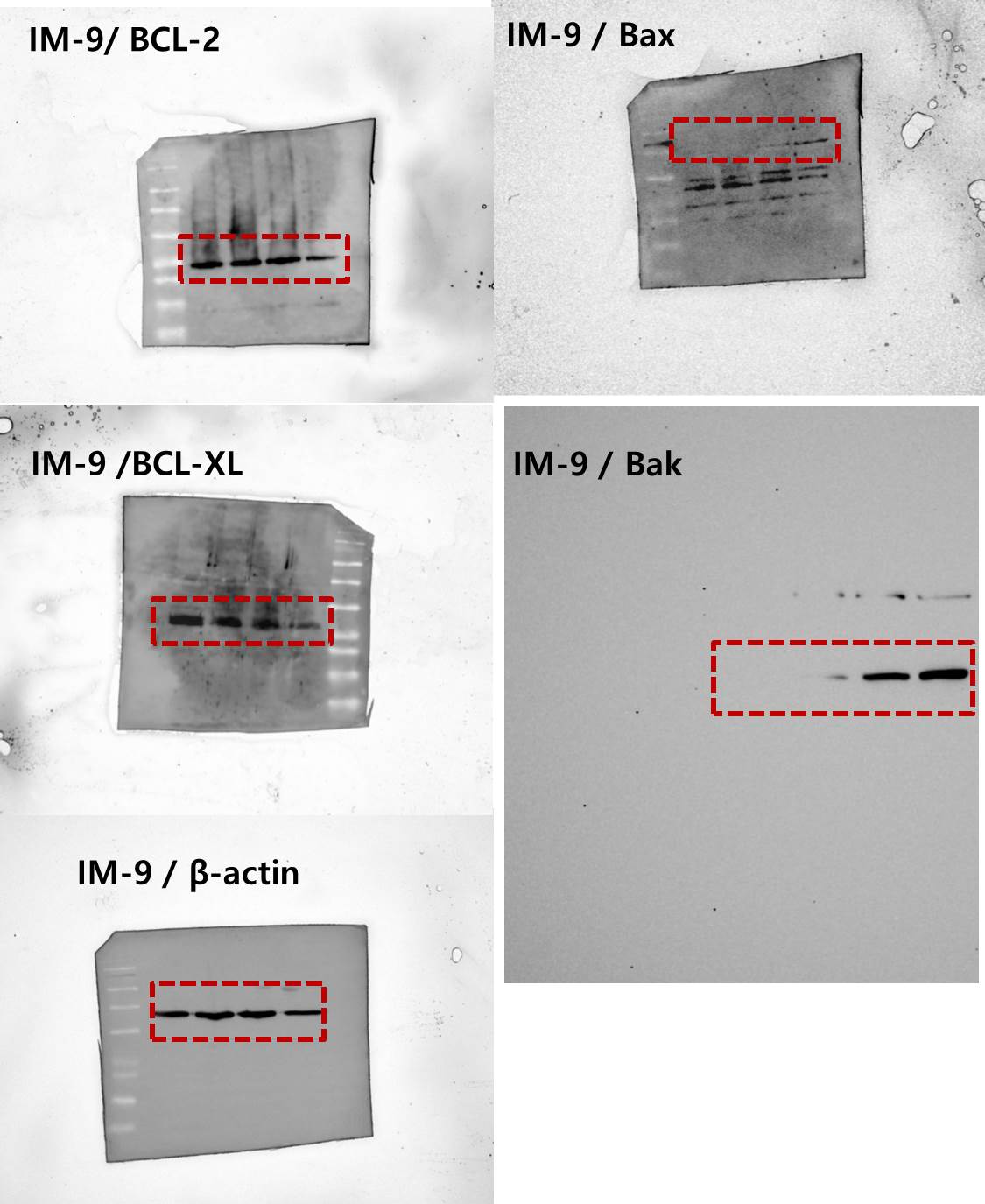
**

**Supplementary Figure 8. continued**


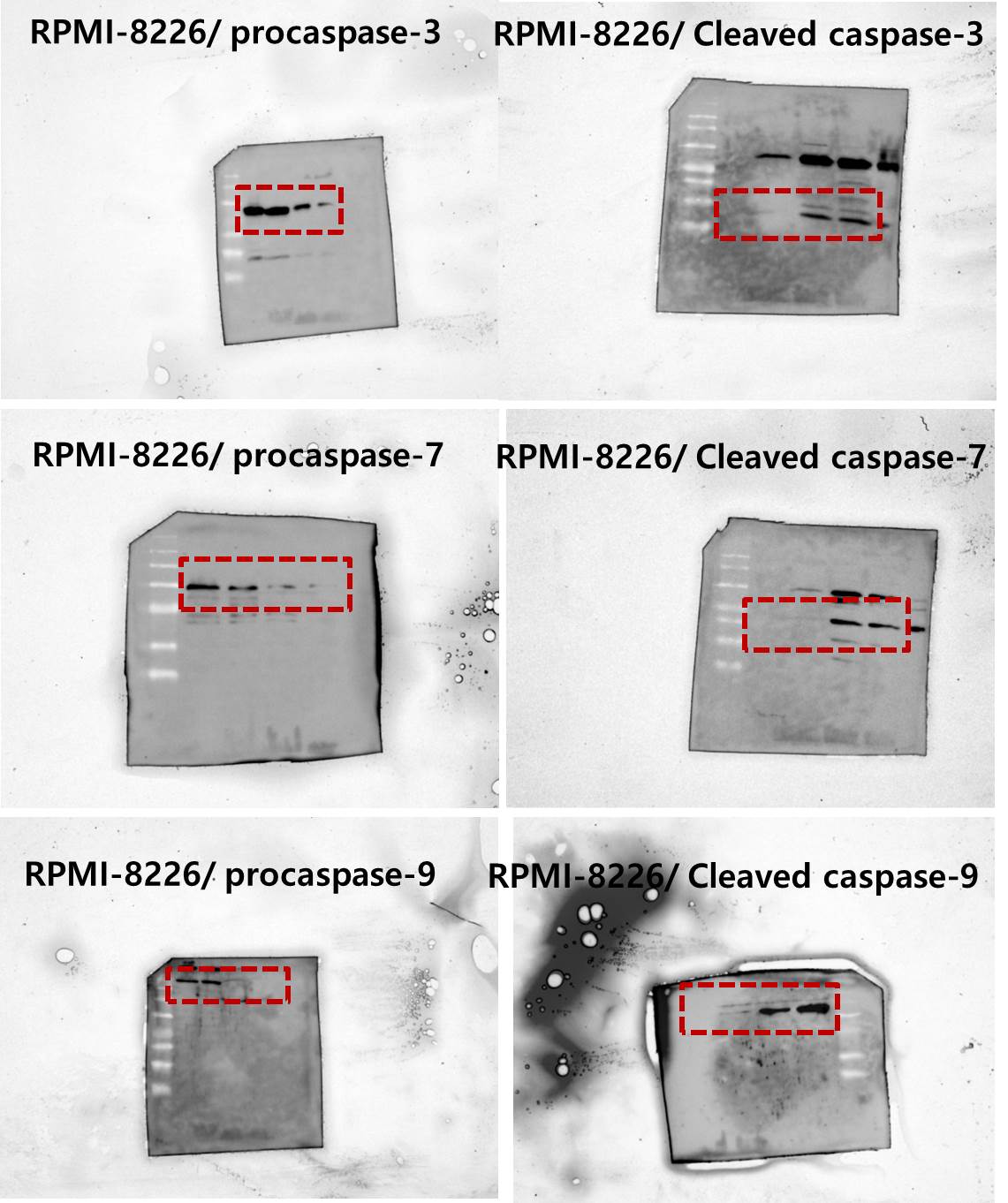


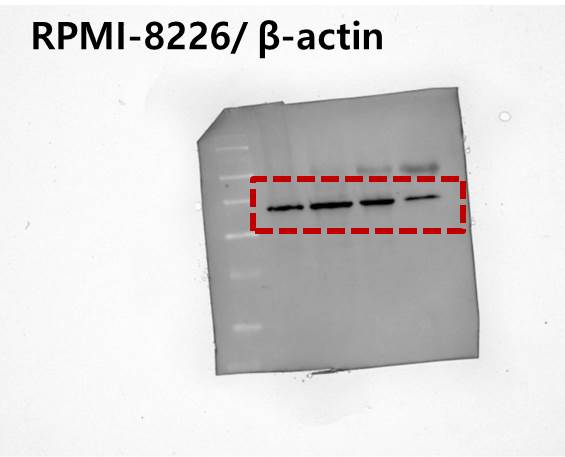


**Supplementary Figure 9.** Original western blots used for **Fig. 5A and 5B**. The blots were developed using the ChemiDoc Touch Imaging System, and analyzed with the Image Lab Software. The red boxes indicate the cropped regions used in the representative figures.

**
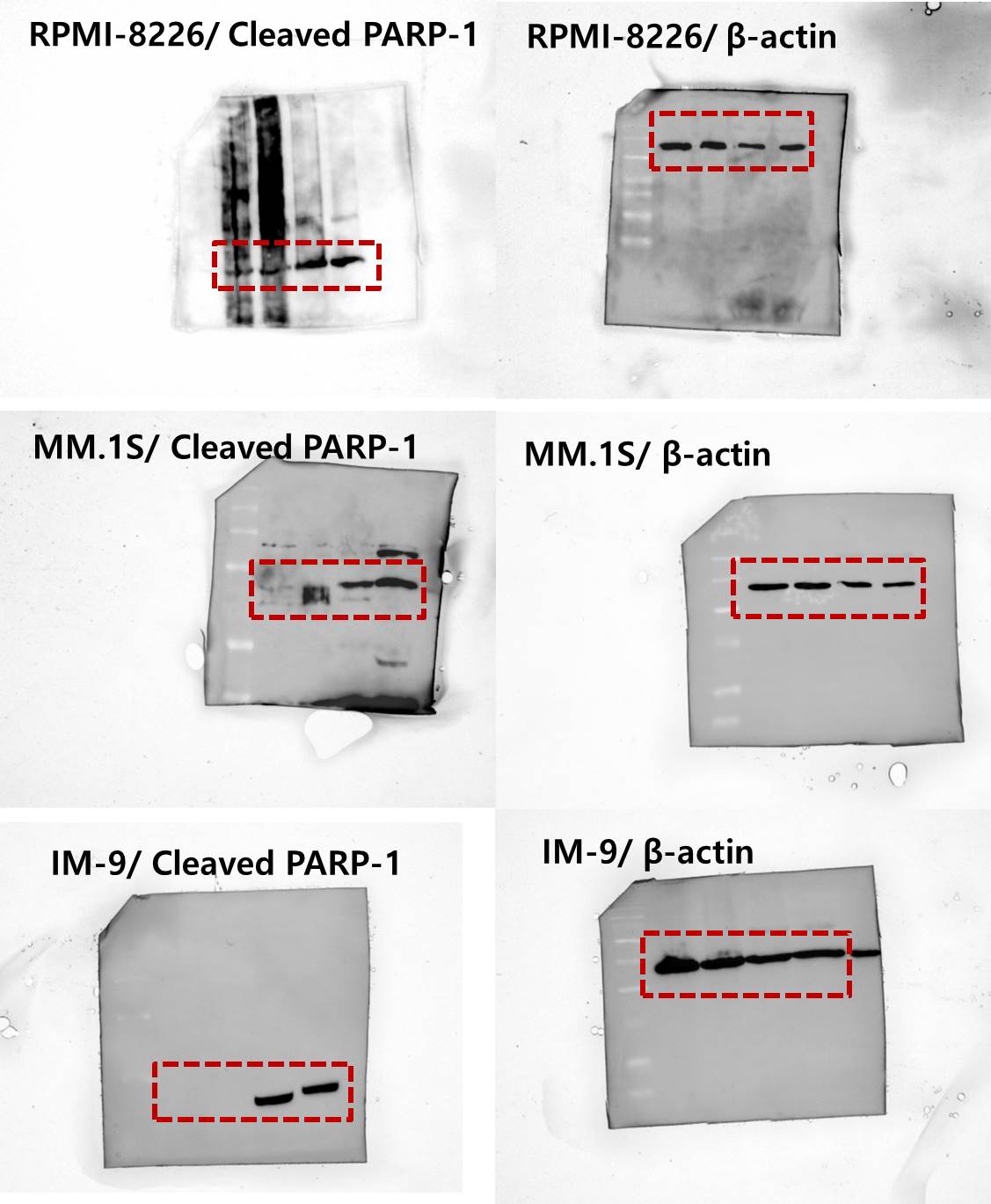
**

**Supplementary Figure 9. continued**

**
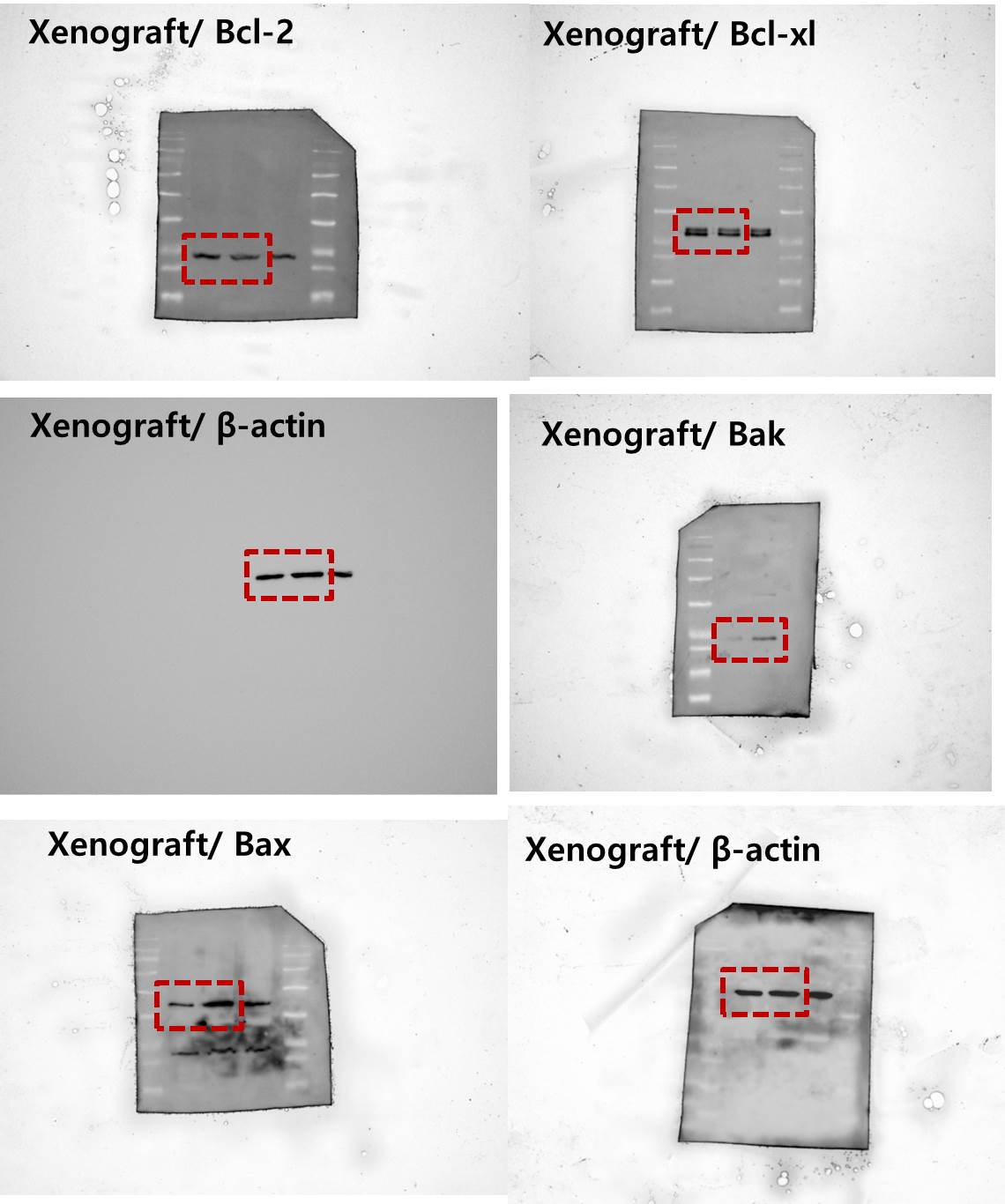
**

**Supplementary Figure 10.** Original western blots used for **Fig. 7E and 7F**. The blots were developed using the ChemiDoc Touch Imaging System, and analyzed with the Image Lab Software. The red boxes indicate the cropped regions used in the representative figures.


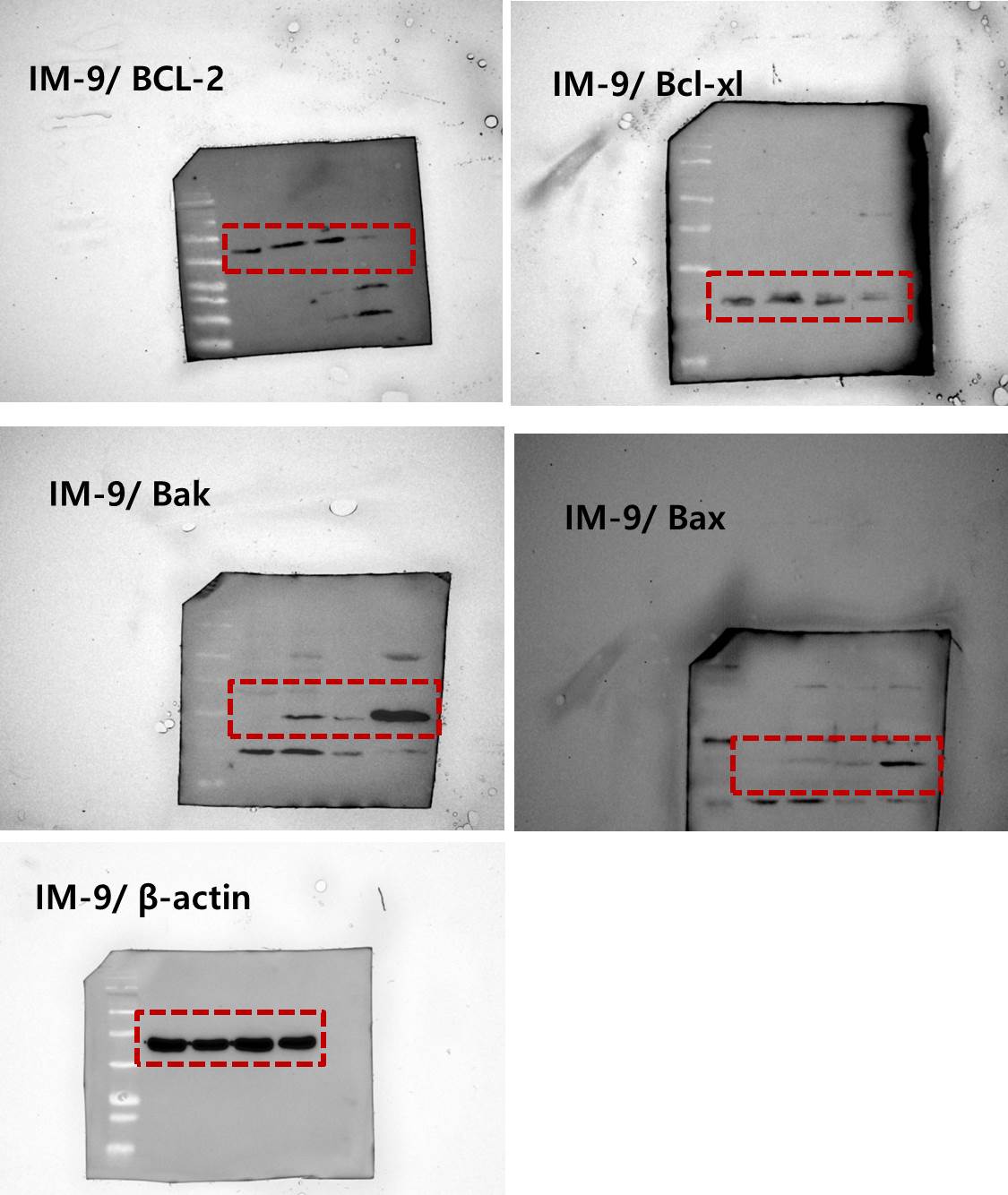


**Supplementary Figure 11.** Original western blots used for **Supplementary Fig. 2.** The blots were developed using the ChemiDoc Touch Imaging System, and analyzed with the Image Lab Software. The red boxes indicate the cropped regions used in the representative figures.

**
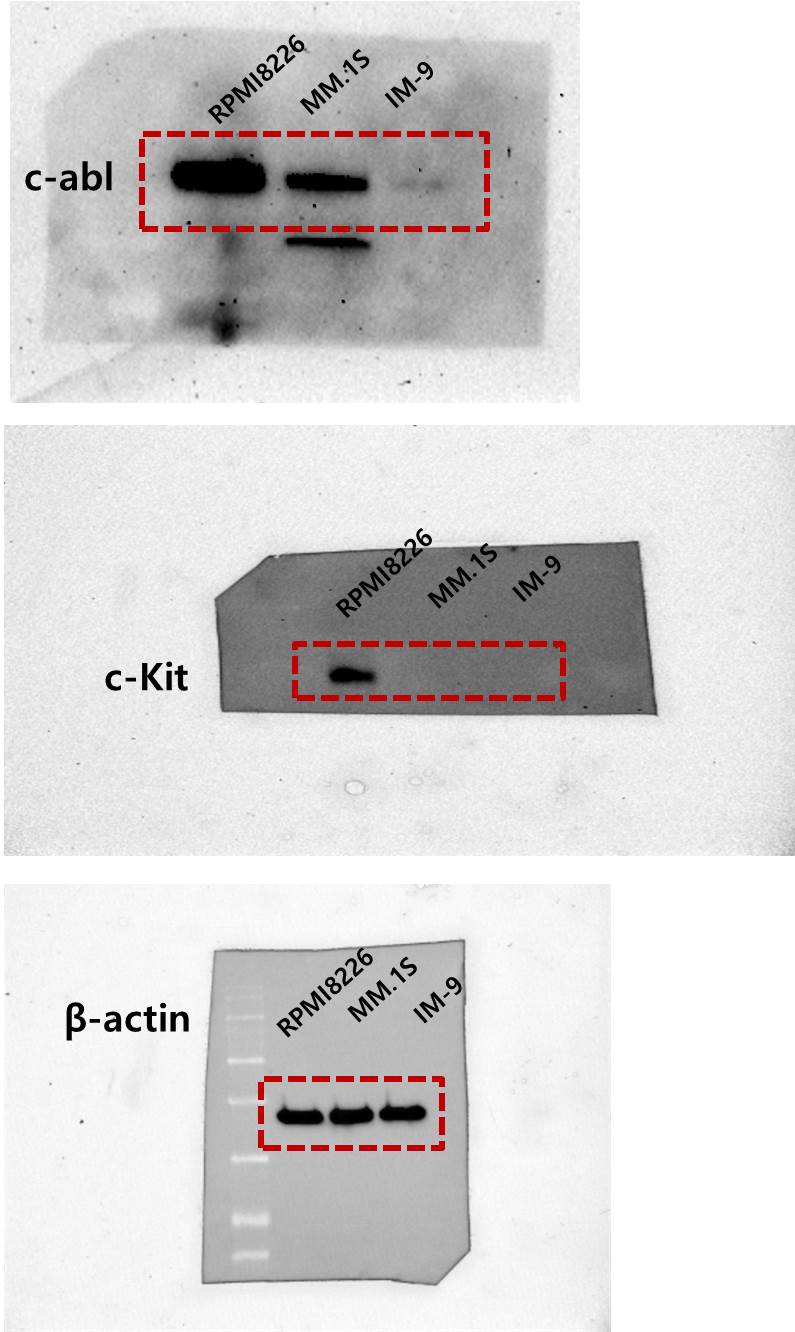
**

**Supplementary Figure 12.** Original western blots used for **Supplementary Fig. 3**. The blots were developed using the ChemiDoc Touch Imaging System, and analyzed with the Image Lab Software. The red boxes indicate the cropped regions used in the representative figures.

**Supplementary Table 2.** Supplementary Methods.

| **Experiments** | **Number of mice** |
| --- | --- |
| *In vivo* test of Tumor cell growth | 15 mice total:  5 mice transplanted with RPMI-8226 cells tumor cell with 1×10^7^  5 mice transplanted with RPMI-8226 cells tumor cell with 2×10^7^  5 mice transplanted with RPMI-8226 cells tumor cell with 3×10^7^ |
| *In vivo* experiment of radotinib dosage determination | 40 mice total:  20 mice treated with the vehicle (0.25% DMSO in DW, intraperitoneally)  20 mice treated with each of 100 mg/kg radotinib, intraperitoneally |
